# Salvianolic Acid a Disrupts the HSP90α‐AKT‐PERK Ternary Complex to Alleviate Atherosclerosis by Activating Endoplasmic Reticulum Stress of Senescent Vascular Smooth Muscle Cells

**DOI:** 10.1002/advs.77891

**Published:** 2026-09-27

**Authors:** Xiuya Guan, Mengkai Lu, Xinhai Cui, Dawei Zhang, Lei Zhang, Lin Lin, Yuan Li, Yuanlong Hu, Chao Li

**Affiliations:** ^1^ College of Traditional Chinese Medicine Shandong University of Traditional Chinese Medicine Jinan China; ^2^ First Clinical Medical College Shandong University of Traditional Chinese Medicine Jinan China; ^3^ Innovation Research Institute of Traditional Chinese Medicine Shandong University of Traditional Chinese Medicine Jinan China

**Keywords:** AKT‐PERK, atherosclerosis, endoplasmic reticulum‐mitochondria contacts, HSP90α, senescence

## Abstract

Atherosclerosis is a chronic inflammatory disease driven by senescent vascular smooth muscle cells (VSMCs). Salvianolic acid A (SAA) exhibits cardiovascular protective effects; however, its role in atherosclerosis remains unclear. This study aimed to investigate the effects and targets of SAA in atherosclerosis. In vitro studies were performed in senescent VSMCs with heat shock protein 90 alpha (HSP90α) overexpression or knockdown, with or without SAA treatment. In vivo, ApoE^−/−^ mice fed a high‐fat diet were treated with SAA and adeno‐associated virus‐mediated VSMC‐specific HSP90α overexpression or knockdown. SAA promoted apoptosis in senescent VSMCs by inhibiting HSP90α. This inhibition promoted ubiquitin‐proteasome‐dependent degradation of protein kinase B (AKT) and activation of PRKR‐Like Endoplasmic Reticulum Kinase (PERK) signaling. SAA increased endoplasmic reticulum‐mitochondrial contacts and mitochondrial calcium uptake. These effects were reversed by HSP90α overexpression. In ApoE^−/−^ mice, SAA reduced the burden of senescent VSMCs, decreased the size of plaque, and increased the stability of the fibrous cap. VSMC‐specific HSP90α knockdown mimicked the protective effects of SAA, whereas HSP90α overexpression reversed SAA‐mediated effects on disease. SAA reduces the burden of senescent VSMCs in atherosclerosis by inhibiting the scaffold function of HSP90α which maintains the AKT‐PERK interaction, to reactivate PERK signaling, thereby alleviating disease progression.

## Graphical abstract

Salvianolic acid A (SAA) selectively induces senescent vascular smooth muscle cell apoptosis, alleviating atherosclerosis. Mechanistically, SAA targets heat shock protein 90 alpha (HSP90α) to disrupt the protein inase B (AKT)‐PRKR‐Like Endoplasmic Reticulum Kinase (PERK) scaffold, driving ubiquitin‐proteasome‐dependent AKT degradation. This pathway reactivates PERK signaling, enhances endoplasmic reticulum‐mitochondria contact area, and increases mitochondrial calcium uptake, offering a promising senolytic approach for cardiovascular protection.

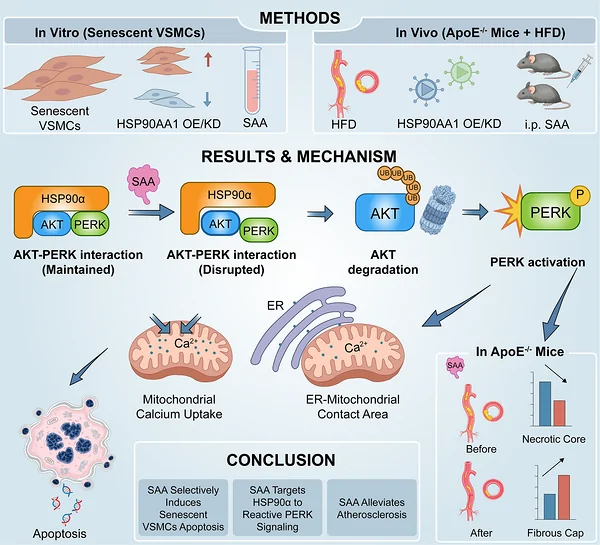

## 1 Introduction

Atherosclerosis has traditionally been defined as a lipid‐driven chronic inflammatory disease [1]. Over the past few decades, even though statins have significantly reduced the morbidity and mortality of atherosclerosis, the incidence of residual cardiovascular risk has remained high [2, 3]. With recent advances in single‐cell lineage tracing, a new consensus is emerging that atherosclerosis is fundamentally a vascular smooth muscle cell (VSMC)‐driven tumor‐like disease [4]. Similar to tumor cells, VSMCs within plaques undergo clonal expansion, dedifferentiation, and metabolic reprogramming, forming pathological structures akin to solid tumors. Within plaques, senescent VSMCs accumulate and mimic the behavior of drug‐resistant, dormant tumor cells. These cells persist long‐term and actively remodel the plaque microenvironment through the senescence‐associated secretory phenotype (SASP), driving necrotic core expansion and fibrous cap thinning [5, 6]. Consequently, developing therapeutic strategies that specifically target this tumor‐like survival mechanism to eliminate senescent VSMCs represents a pivotal strategy for plaque stabilization.

Senescent VSMCs are characterized by high secretion of SASP and resistance to apoptosis, which in turn contribute to survival and accumulation in the altered plaque microenvironment [7, 8]. They propagate senescence to surrounding non‐senescent cells through SASP [9]. Studies have shown that selective elimination of senescent VSMCs can preserve the contractile properties of the remaining VSMCs and attenuate the progression of disease [10, 11]. Senolytic agents, which selectively eliminate senescent cells by targeting the survival mechanisms, have shown remarkable potential in extending lifespan in animals [12]. In oncology, heat shock protein 90 alpha (HSP90α) has been recognized as a critical scaffold supporting cell survival [13]. Given the parallels between tumorigenesis and atherogenesis, we hypothesize that senescent VSMCs may similarly hijack this HSP90α‐mediated machinery to maintain cellular homeostasis and evade cell death. Specifically, the potential role of HSP90α in restraining the full activation of endoplasmic reticulum stress (ERS) suggests a mechanism by which these pathogenic cells could sustain a metastable survival state despite the harsh plaque microenvironment. However, the precise interplay between HSP90α and ERS‐mediated apoptosis in the context of vascular senescence remains largely unexplored.

In this study, we observed that senescent VSMCs in atherosclerotic plaques exhibit incomplete activation of the ERS, which enables these cells to preserve SASP metabolic activity. HSP90α maintains the interaction between Protein Kinase B (AKT) and PRKR‐Like Endoplasmic Reticulum Kinase (PERK) in senescent VSMCs, thereby promoting the survival of senescent VSMCs. Salvianolic acid A (SAA), a bioactive component derived from traditional Chinese medicine, was identified as a potent inhibitor of HSP90α, thereby activating ERS.

## 2 Methods

### 2.1 Human Atherosclerotic Plaques

Human atherosclerotic plaques were obtained from patients who underwent carotid endarterectomy. All samples were collected using a protocol that was approved by the Ethics Committee of the Affiliated Hospital of Shandong University of Traditional Chinese Medicine (Approval No. 2025‐034‐KY), and informed consent was obtained from all patients.

### 2.2 Animals

All experimental mice were purchased from Vital River Laboratory Animal Technology Co., Ltd (Beijing, China), including 8‐week‐old male ApoE^−/−^ mice (C57BL/6 background) and 8‐week‐old male Wild‐type (WT; C57BL/6 background) mice. All mouse experimental procedures were performed strictly in accordance with the United States Public Health Services Guide for the Care and Use of Laboratory Animals. All experimental protocols were approved by the Animal Care and Use Committee of the School of Life Sciences and Technology, Shandong University of Traditional Chinese Medicine (Approval No. SDUTCM20241112001). Based on standard power calculations (α = 0.05 and power = 0.8), the number of mice used was sufficient to yield statistically significant differences [14, 15]. All mice were randomly assigned to experimental groups at the initiation of high‐fat diet (HFD) feeding. Quantification of aortic root plaque area, lipid deposition, and plaque stability markers was performed by an experienced pathologist blinded to the group allocation of all specimens. No mice fed a HFD were excluded from final analyses.

All experimental mice were group‐housed at a temperature of 22°C ± 2°C, under a 12‐h light‐dark cycle, and fed ad libitum with free access to water. All ApoE^−/−^ mice were fed a HFD (containing 21% fat and 0.5% cholesterol; Beijing KeAo XieLi Feed Co., Ltd.), whereas all WT mice received a standard chow diet as controls. Hsp90aa1 overexpression adeno‐associated virus (AAV) (HBAAV2/9‐Sm22a‐m‐Hsp90aa1‐3xflag‐ZsGreen), Hsp90aa1 knockdown AAV (HBAAV2/9‐Sm22a‐mir30‐1/2‐m‐Hsp90aa1‐ZsGreen), and their respective control vectors were constructed by HanBio Technology Co., Ltd. (Han Bio, Shanghai, China).

ApoE^−/−^ mice were randomized to receive normal saline (vehicle control) or SAA (5, 10, and 20 mg/kg; Shanghai Yuanye Bio‐Technology Co., Ltd) via intraperitoneal (IP) injection once daily after 8 weeks of HFD. WT mice served as controls and received normal saline by IP injection on the same schedule. After 12 weeks, the mice were weighed and anesthetized with isoflurane (Shandong Ante Animal Husbandry Technology Co., Ltd.; Veterinary Drug Approval No. 151987015). Serum was obtained by centrifuging the blood collected from the orbital venous plexus at 3000 rpm and 4°C for 15 min. After collection of tissues, the heart, aorta, liver, and kidneys were fixed in 4% paraformaldehyde. All remaining tissues were stored at −80°C until further use.

### 2.3 Cell Culture

Human aortic vascular smooth muscle cells (HAVSMCs) were purchased from ScienceCell Research Laboratories (San Diego, USA). Cells were cultured in Dulbecco's modified Eagle medium (DMEM; Gibco, China) containing 10% fetal bovine serum (FBS; Shanghai Shuangru Biology Science & Technology Co., Ltd.) and 1% penicillin/streptomycin, with the culture environment set as a humidified incubator at 37°C and 5% CO_2_.

A stress‐induced premature senescence model was developed essentially as previously described [10]. Palmitic acid‐conjugated bovine serum albumin (BSA) was applied to mimic the lipotoxic stress occurring during atherosclerosis. Briefly, VSMCs were treated with 200 µm palmitate (PA) complexed with 10% BSA (PA‐BSA) for 48 h, followed by incubation in fresh medium for an additional 48 h (senescence induction group). As a control, VSMCs treated with 10% BSA dissolved in phosphate‐buffered saline (PBS; PBS‐BSA control group) were subjected to the same 48‐h treatment and 48‐h fresh medium incubation schedule. A stress control group was set up by treating VSMCs with PA‐BSA for 48 h without the subsequent incubation with fresh medium and defined as stressed VSMCs.

The preparation process of PA‐BSA was as follows: Sodium PA (Cat. No.: P9830, Solarbio) was dissolved in sterile PBS by heating to 70°C with constant stirring until completely dissolved. This solution was then mixed with acid‐free BSA (Cat. No.: A8850, Solarbio) and incubated in a 50°C water bath for 30 min to form PA‐BSA complexes. After cooling to room temperature, the mixture was sterilized by filtration through a 0.22 µm sterile filter. The BSA‐PBS control was prepared by dissolving 10% acid‐free BSA in sterile PBS, followed by the same filtration sterilization step.

The lentiviral overexpression plasmid for HSP90AA1 was constructed using the pHBLV‐CMV‐MCS‐3FLAG‐EF1‐T2A‐Puro vector, with the corresponding empty vector used as the control. Plasmids were transiently transfected into HAVSMCs using LipoFiter 3.0 Liposome Transfection Reagent (Cat. No.: HB‐LF3‐1000, Hanheng Biotechnology Co., Ltd., Shanghai, China) following the manufacturer's instructions. The small interfering RNA (siRNA) sequences are as follows: human PERK: 5’‐GAG UUG UUU UUG AAG CUA ATT, human HSP90AA1: 5’‐CGU GAG AUG UUG CAA CAA ATT, huaman mitochondrial calcium uniporter (MCU): 5’‐GCU GGU CAU UAA UGA CUU ATT, and random control sequence: 5’‐UUC UCC GAA CGU GUC ACG UTT. siRNA duplexes were transfected into HAVSMCs using RNAFit Reagent (Cat. No.: HB‐RF‐1000, Hanheng Biotechnology Co., Ltd., Shanghai, China) in accordance with the manufacturer's protocol.

After establishing the senescent cell model, the cells were treated with SAA. The SAA was dissolved in dimethyl sulfoxide (DMSO) to prepare a 10 mm stock solution, then diluted with cell culture medium to final concentrations of 20, 60, and 100 µm; the final concentration of DMSO in the medium was maintained at ≤0.5%. Vehicle control groups received an equal volume of DMSO. Thapsigargin was dissolved in PBS to prepare a 100 µm stock solution, and ruthenium red was dissolved in PBS immediately before use. Both solutions were sterilized by filtration through a 0.22 µm sterile filter and further diluted with cell culture medium to final concentrations of 0.1 and 4 µm for thapsigargin and ruthenium red, respectively; cells in the vehicle control groups for these two drugs were administered an equivalent volume of PBS. SAA and thapsigargin treatments involved incubation for 24 h, and ruthenium red treatments were performed for 6 h. All treatments were performed under standard culture conditions (37°C, 5% CO_2_, humidified atmosphere).

### 2.4 CCK‐8 Assay

Normal VSMCs and senescent VSMCs in the logarithmic growth phase were harvested. The cell density was adjusted to 1 × 10^4^ cells/well with DMEM containing 10% FBS, and the cells were seeded into 96‐well cell culture plates with a final volume of 100 µL per well. The culture plate was incubated at 37°C in a humidified 5% CO_2_ incubator for 24 h. The original medium was discarded, and fresh medium containing different concentrations of the drug was added to respective wells, with six replicate wells assigned per group. The blank control group received an equal volume of drug‐free medium. After drug treatment, 10 µL of CCK‐8 assay reagent (Cat. No.:C0037, Beyotime Biotechnology) was added to each well. The culture plate was gently shaken to ensure thorough mixing of the reagent with the medium, followed by further incubation for 4 h. A microplate reader was used to measure the absorbance of each well at a wavelength of 450 nm.

### 2.5 Proteomics Analysis

Senescent VSMCs were rinsed with pre‐cooled PBS and then lysed on ice using RIPA lysis buffer supplemented with protease and phosphatase inhibitors, followed by centrifugation to obtain total protein. The protein concentration was quantified via the BCA assay, and the extraction quality was verified by sodium dodecyl dulfate (SDS)‐polyacrylamide gel electrophoresis (SDS‐PAGE). An appropriate amount of protein was subjected to reduction with dithiothreitol, alkylation with Iodoacetamide, and then digestion with trypsin. The resulting peptides were desalted, lyophilized, reconstituted, and separated by reversed‐phase high‐performance liquid chromatography prior to detection by tandem mass spectrometry. Differentially expressed proteins were subsequently identified.

### 2.6 Integrated Analysis of a Human Single‐Cell Atherosclerosis Atlas

Raw count matrices of 40 samples from four published studies were integrated, with all data obtained from the Gene Expression Omnibus and Zenodo public databases [16, 17, 18, 19]. The studies covered four groups of human atherosclerotic lesion tissues, including carotid artery lesion samples collected via endarterectomy and coronary artery lesion samples dissected from explanted hearts of heart transplant recipients. VSMCs were annotated according to established marker genes, including actin alpha 2, smooth muscle (ACTA2) and transgelin (TAGLN). The Harmony algorithm, implemented with the R package “harmony,” was applied to integrate multiple datasets and remove batch effects, thereby enabling direct comparison of cellular data derived from different studies [20].

### 2.7 Western Blot

Proteins were extracted using cold RIPA lysis buffer supplemented with protease inhibitor cocktail and phosphatase inhibitor cocktail. The lysates were mixed with protein loading buffer and heated to denature the proteins. Equal amounts of the denatured proteins were separated by SDS‐PAGE and transferred to polyvinylidene fluoride membranes for subsequent analysis.

The membranes were blocked for 1 h with 5% non‐fat dry milk in tris‐buffered saline with tween 20 and immunoblotted with primary antibodies recognizing cyclin‐dependent kinase inhibitor 2A (p16, Cat. No.: 18769S, Cell Signaling Technology), cyclin‐dependent kinase inhibitor 1A (p21, Cat. No.: 82669‐2‐RR, Proteintech), HSP90α (Cat. No.: 60318‐1‐Ig, Proteintech), AKT (Cat. No.: 4691S, Cell Signaling Technology), PERK (Cat. No.: 68482‐1‐Ig, Proteintech), phosphorylated‐PERK (P‐PERK, Cat. No.: 82534‐1‐RR, Proteintech), eukaryotic translation initiation factor 2 subunit alpha (eIF2α, Cat. No.: 11170‐1‐AP, Proteintech), phosphorylated‐eIF2α (P‐eIF2α, Cat. No.: 68023‐1‐Ig, Proteintech), C/EBP homologous protein (CHOP, Cat. No.: 15204‐1‐AP, Proteintech), branched ubiquitin antibody sampler kit (Cat. No.: 33959T, Cell Signaling Technology), Flag (Cat. No.: 20543‐1‐AP, Proteintech), MCU (Cat. No.: 26312‐1‐AP, Proteintech), activating transcription factor 6 (ATF6, Cat. No.: 24169‐1‐AP, Proteintech), Phosphorylated Inositol‐Requiring Enzyme 1 (p‐IRE1, Cat. No.: AP0878SP, ABclonal), IRE1 (Cat. No.: 27528‐1‐AP, Proteintech), or β‐actin (Cat. No.: 66009‐1‐Ig, Proteintech). All primary antibodies were applied with overnight incubations at 4°C, followed by incubation with corresponding secondary antibodies at room temperature for 1 h. The secondary antibodies used were horseradish peroxidase (HRP)‐conjugated goat anti‐rabbit IgG (H+L) (Cat. No.: GB23303, Servicebio) and HRP‐conjugated goat anti‐mouse IgG (H+L) (Cat. No.: GB23301, Servicebio).

### 2.8 Co‐Immunoprecipitation and Ubiquitination Detection

Protein‐protein interactions were detected using a co‐immunoprecipitation kit (P2179M, Beyotime Biotechnology). After lysis with inhibitor‐containing buffer, samples were mixed with an anti‐HSP90α antibody (Cat. No.: 60318‐1‐Ig, Proteintech) or anti‐AKT antibody (Cat. No.: 4691S, Cell Signaling Technology) bound to protein A+G magnetic beads and incubated overnight at 4°C. The magnetic beads were separated by magnetic force, then washed 3 times with pre‐cooled lysis buffer to remove unbound proteins. The magnetic beads were resuspended in 1× loading buffer and incubated at 95°C for 5 min. After magnetic separation for 10 s, the supernatant was collected for subsequent Western blotting.

For ubiquitination analysis, cells were pretreated with 50 µm proteasome inhibitor MG132 for 4 h. Cells were subjected to co‐immunoprecipitation with the anti‐AKT antibody in a lysis buffer system containing 0.1% SDS, followed by detection via Western blotting.

### 2.9 Domain Mapping

VSMCs were transfected with plasmids encoding the N‐terminal domain (amino acids 1–284), middle domain (amino acids 285–620), and C‐terminal domain (amino acids 621–732) of HSP90AA1 or empty vector before total protein extraction. Subsequent steps were performed according to the standard co‐immunoprecipitation protocol. HSP90AA1 plasmids were purchased from Hanheng Biotechnology.

### 2.10 Immunofluorescence Staining

Cells were fixed with 4% paraformaldehyde for 20 min and washed three times with PBS. Subsequently, they were permeabilized with Triton X‐100 (Cat. No.: P0096, Beyotime) for 30 min, followed by blocking with 5% BSA (Cat. No.: A8020, Solarbio) for 30 min. The cells were then incubated overnight at 4°C with the following primary antibodies: HSP90α (Cat. No.: 60318‐1‐Ig, Proteintech) and CHOP (Cat. No.: 15204‐1‐AP, Proteintech). The corresponding secondary antibodies were a fluorescein isothiocyanate (FITC)‐conjugated goat anti‐rabbit IgG (Cat. No.: GB22303, Servicebio) and a Cy3‐conjugated goat anti‐mouse IgG (H+L) (Cat. No.: GB21301, Servicebio). The secondary incubation was performed for 1 h at room temperature.

Actin filaments were stained with SF633‐phalloidin (Cat. No.: CA1670, Solarbio) for 30 min, and cell nuclei were counterstained with DAPI (Cat. No.: C1005, Beyotime) for 30 min. Multiplex fluorescence staining was performed with a kit according to the manufacturer's instructions (Cat. No.: P1351S, Beyotime). PERK was detected using a Tyramide‐488 conjugate (Cat. No.: 20582‐1‐AP, Proteintech), AKT with Tyramide‐555(Cat. No.: 4691S, Cell Signaling Technology), and HSP90α with Tyramide‐647 (Cat. No.: 60318‐1‐Ig, Proteintech). Images were captured using an epifluorescence microscope (ZEISS, Germany).

Plaque tissues and mouse aortas were fixed in 4% paraformaldehyde at 4°C for 24 h. After being washed three times with PBS, the tissues were incubated overnight in 30% sucrose solution at 4°C for cryoprotection. The tissues were embedded in Optimal Cutting Temperature compound for cryosectioning, and the resulting sections were stored at −80°C until use. Prior to staining, the frozen sections were first thawed at room temperature for 20 min to remove condensation and recover antigens, followed by antigen retrieval.

The sections were blocked with 5% serum for 1 h at room temperature. After three washes with PBS, the sections were incubated overnight at 4°C with the following primary antibodies: p21 (Cat. No.: 82669‐2‐RR, Proteintech), HSP90α (Cat. No.: 60318‐1‐Ig, Proteintech), CHOP (Cat. No.: 15204‐1‐AP, Proteintech), or Alpha‐Smooth Muscle Actin (α‐SMA, Cat. No.: 14395‐1‐AP, Proteintech). After washing, the sections were incubated with appropriate secondary antibodies and counterstained with DAPI.

### 2.11 Endoplasmic reticulum (ER) Hematoxylin and Eosin‑Mitochondria Contacts Detection

The ER was labeled with ER‑Tracker Blue‑White DPX (Cat. No. E12353, Thermo Fisher Scientific), and mitochondria were labeled with MitoTracker Deep Red FM (Cat. No. M22426, Thermo Fisher Scientific). Cells were incubated with each probe at the concentrations recommended by the manufacturer, and the incubation time for each probe did not exceed 30 min.

### 2.12 Biotin Pull‐Down Assay

For the pull‐down assay, cell lysates were incubated with biotin‐conjugated SAA (bio‑SAA) in the absence or presence of excess unlabeled SAA at 4°C overnight. The protein complexes were then captured with streptavidin‑agarose beads for 6 h. The bound proteins were then analyzed by Western blotting with anti‑HSP90α.

### 2.13 Cross‐Linking Assay

For cross‑linking experiments, cells were incubated with disuccinimidyl suberate (DSS, Cat. No. A39267, Thermo Fisher Scientific) for 30 min at room temperature. The reaction was terminated with Tris‑HCl (50 mm, pH 7.5) for 15 min. Cell lysates were then subjected to SDS‑PAGE and immunoblotted with specific antibodies.

### 2.14 Hematoxylin and Eosin (H&E) and Masson's Staining

Following transcardiac perfusion via the left ventricle with ice‐cold PBS to flush residual blood, the hearts were immediately dissected and fixed in 4% paraformaldehyde at 4°C for 24 h. After fixation, the tissues were subjected to gradient dehydration through a graded ethanol series, cleared in xylene, and subsequently embedded in paraffin. Serial sections (5 µm thick) were prepared and mounted on poly‐L‐lysine‐coated glass slides. H&E (Cat. No.: G1076, Servicebio) staining and MASSON trichrome staining (Cat. No.: G1006, Servicebio) were performed strictly according to the manufacturers' protocols.

### 2.15 Oil Red O Staining

Following transcardiac perfusion with ice‐cold PBS, the entire aorta from the aortic root to the iliac bifurcation was carefully dissected, and the peri‐adventitial fat tissue was removed under a stereomicroscope. Fresh aortas were rinsed with PBS to eliminate residual blood, then dehydrated in 60% isopropanol for 10 min. Oil red O staining (Cat. No.: G1015, Servicebio) was performed in accordance with the manufacturer's protocol. Briefly, the aortas were immersed in freshly prepared Oil red O working solution and incubated at 37°C for 30 min, followed by differentiation with 60% isopropanol for 30 s to eliminate non‐specific background staining. After rinsing with distilled water to terminate the reaction, the aortas were mounted on glass slides with glycerol gelatin mounting medium (Cat. No.: G1402, Servicebio) to prevent desiccation. The stained gross tissues were flattened and spread out on a black background board. Under optimal lighting conditions, the focal length was adjusted prior to image acquisition.

### 2.16 Senescence‐Associated β‐Galactosidase (SA‐β‐gal) Staining

Cellular senescence was detected using a SA‐β‐gal Staining Kit (Cat. No.: C0602, Beyotime) according to the manufacturer's instructions. After being washed three times with PBS, the cells were fixed with 4% paraformaldehyde for 15 min at room temperature. Following fixation, the cells were washed three times with PBS to remove residual paraformaldehyde. The cells were incubated with the staining working solution at 37°C overnight, protected from light. After incubation, the cells were washed twice with PBS to eliminate unbound staining solution. Senescent cells, distinguished by blue staining, were observed and imaged under a light microscope.

### 2.17 Apoptosis Staining

Apoptotic cells were assessed using a Mitochondrial Membrane Potential and Apoptosis Assay Kit (Cat. No.: C1071M, Beyotime) as per the manufacturer's protocol. The cells were first incubated with Mito‐Tracker Red CMXRos at 37°C for 30 min in the dark to permit monitoring of mitochondrial membrane potential. After two gentle washes with PBS, the cells were incubated with Annexin V‐FITC at room temperature for 30 min in the dark to label apoptotic cells. Nuclei were counterstained with Hoechst 33342 for 5 min at room temperature, protected from light. The cells were washed twice with cold PBS. Images were captured using an epifluorescence microscope (ZEISS, Germany).

### 2.18 Detection of Mitochondria‐Associated ER Membranes

Cells were gently rinsed three times with pre‐warmed PBS to remove residual medium. Cells were then detached using a cell scraper and centrifuged at 800 × g for 5 min at 4°C to form a cell pellet. The cell pellet was immediately immersed in 2.5% glutaraldehyde and resuspended by pipetting to disperse clumps. Cells were fixed at room temperature in the dark for 30 min to preserve ultrastructural integrity. This was followed by dehydration through a graded ethanol series for 15 min per step and two subsequent washes with 100% acetone. The samples were then infiltrated with a graded series of acetone and Epon 812 resin (Cat. No.: GP2001, Servicebio; 1:1 acetone/resin for 2 h at room temperature followed by 1:3 acetone/resin overnight) before being embedded in pure Epon 812 resin for polymerization at 60°C for 48 h. Ultra‐thin sections (60–80 nm) were cut and stained sequentially with 2% uranyl acetate for 15 min and lead citrate for 10 min in the dark. Finally, the sections were observed using a Hitachi transmission electron microscopy (TEM) system operated at an accelerating voltage of 80 kV, and images of mitochondria and ER were captured.

### 2.19 Fluorescence Detection of Mitochondrial Calcium

Mitochondrial calcium levels were measured using a Mitochondrial Calcium Assay Kit (Cat. No.: S1062S, Beyotime) according to the manufacturer's instructions. Briefly, after washing the cells twice with pre‐warmed PBS, the cells were incubated with Rhod‐2 Acetoxymethyl Ester (Rhod‐2 AM) at 37°C in the dark for 30 min. After probe loading, the cells were washed three times with pre‐warmed PBS. Cell nuclei were counterstained with Hoechst 33342 (Cat. No.: S1026, Beyotime) at room temperature for 5 min in the dark. Images were acquired using an epifluorescence microscope (ZEISS, Germany).

### 2.20 Cellular Thermal Shift Assay

Cells were lysed on ice for 30 min in RIPA buffer supplemented with 1% protease inhibitor cocktail and 1% phosphatase inhibitor cocktail. The lysates were then centrifuged at 12 000 × g for 10 min at 4°C to remove cell debris, and the resulting supernatants were collected as total protein extracts. Equal amounts of protein extracts were incubated with either 100 µm SAA or an equal volume of DMSO (vehicle control) at room temperature for 30 min. The mixture was aliquoted into tubes, which were heated to seven different temperatures (52°C, 55°C, 58°C, 61°C, 64°C, 67°C, and 70°C) for 3 min, followed by cooling at room temperature for 3 min. The samples were then centrifuged at 20 000 × g for 20 min at 4°C. The resulting supernatants were mixed with SDS loading buffer and heated at 95°C for 10 min, after which Western blotting was performed to detect HSP90α that had remained folded.

### 2.21 Cycloheximide (CHX) Chase Assay

The stability of the AKT protein was assessed using a CHX chase assay. Cells were seeded into culture dishes and allowed to adhere overnight at 37°C in a humidified 5% CO_2_ incubator until reaching 70%–80% confluence. CHX was dissolved in DMSO to prepare a 10 mm stock solution, then diluted with cell culture medium to a final concentration of 50 µm, with the final DMSO concentration maintained at ≤0.5%. The cells were treated with 50 µm CHX (Cat. No.: C408230, Aladdin), while the control group was treated with an equal volume of DMSO in culture medium.

### 2.22 Natural Product Dataset Construction

A comprehensive literature search of publications from 1947 to 2024 retrieved a total of 57 290 articles. Review articles were excluded, and only those articles that utilized in vivo and/or in vitro assays were included. Subsequent manual screening of titles and abstracts was performed, with a focus on studies that confirmed the anti‐atherosclerotic activity of the compounds. This process identified a final set of 112 unique compounds. The detailed search strategy and the resulting compound dataset are provided in Tables S1 and S2.

### 2.23 Pharmacophore‐Based Screening and Molecular Docking

First, the molecules in the list of 112 small‐molecule compounds were screened using a pharmacophore library selected from the ePharmaLib database via the Pharao software (available at https://github.com/silicos‐it/pharao) [21, 22]. Three molecular docking programs were employed to conduct molecular docking analyses between the receptor protein (HSP90α) and ligand molecules, specifically AutoDock Vina (version 1.2.0) [23], LeDock (v1.0) [24], and UCSF DOCK (v6.10). Using the crystal structure of HSP90α in complex with the established HSP90α inhibitor N‐dimethylaminoethylamino‐geldanamycin (17‐desmethoxy‐17‐N, 17‐DMAG; PDB ID: 1OSF) as a reference, the docking box parameters were defined as follows: after removing water molecules and the 17‐DMAG ligand, a docking box was delineated to extend 5.0 Å in all three spatial dimensions (x/y/z) from the geometric center of the original 17‐DMAG binding site. This ensured comprehensive coverage of the HSP90α active site channel [25]. The output from each docking method included multiple binding conformations ranked in descending order by their docking scores (higher scores indicate stronger binding affinity); subsequently, the Robust Rank Aggregation [26] method was employed to integrate and re‐rank the scores of conformations from the three docking programs, and the conformation with the highest composite rank was selected for subsequent analysis.

HDOCK was employed to perform protein‐protein docking between HSP90α and target proteins (AKT and PERK) and assess their binding possibility [27]. A confidence score greater than 0.7 indicates a high probability of binding between the two proteins, as defined by the HDOCK server (http://hdock.phys.hust.edu.cn/help.php). The three‐dimensional structures of HSP90α (PDB ID: 7RY1), AKT (PDB ID: 8UW9), and PERK (PDB ID: 4 × 7K) were retrieved from the RCSB Protein Data Bank and were preprocessed by removing water molecules and non‐native ligands prior to docking. Subsequently, the AREA‐AFFINITY tool (available at https://affinity.cuhk.edu.cn/) was used to quantitatively predict the binding affinity [log(K), where K is the dissociation constant] between HSP90α and the target proteins [28].

### 2.24 Statistical Analysis

Data were analyzed using R and RStudio software and are presented as the mean ± standard deviation (mean ± SD). Comparisons among multiple groups were performed using one‐way analysis of variance (one‐way ANOVA). A *p*‐value of less than 0.05 (*p* < 0.05) was considered statistically significant. Image analysis was conducted using ImageJ software (version 1.54p, Maryland, USA) and QuPath software (version 0.5.1, Edinburgh, Scotland).

## 3 Results

### 3.1 Upregulated HSP90α Maintains the Survival of Senescent VSMCs

**FIGURE 1:**
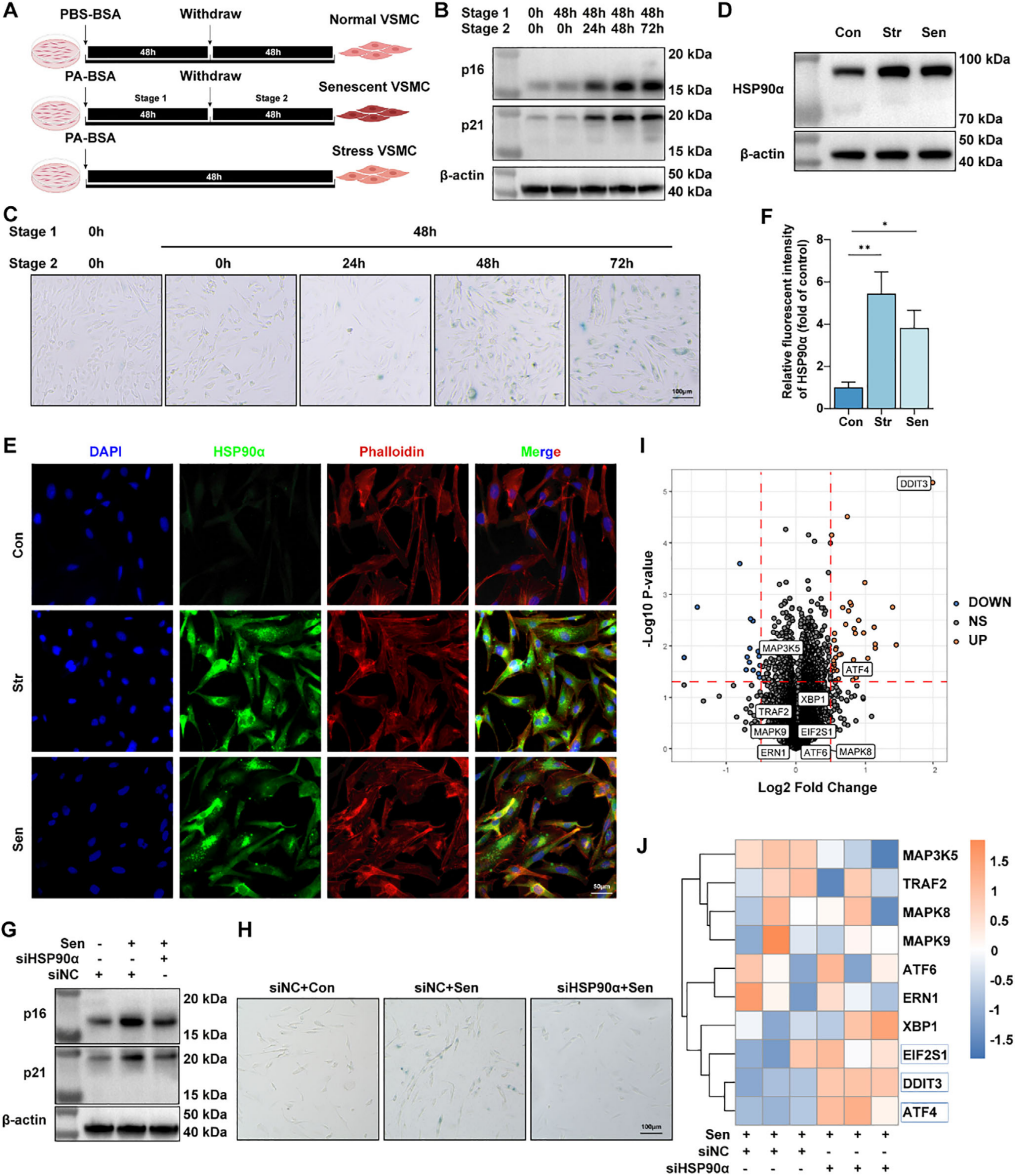
Upregulated HSP90α maintains the survival of senescent VSMCs. (A) Schematic diagram of the experimental models for stressed and senescent VSMCs. (B) Western blot analysis of p16 and p21 protein levels in VSMCs treated with palmitate complexed with 10% bovine serum albumin for 48 h and then recovered in normal medium for the indicated times. (C) Senescence‐associated β‐galactosidase staining of model VSMCs. (D) Western blot analysis of HSP90α protein levels in stressed (Str) and senescent (Sen) VSMCs. (E) Representative immunofluorescence images of HSP90α (green) and DAPI‐stained nuclei (blue) in stressed and senescent VSMCs. (F) Quantification of HSP90α (green) from images as in (E) (*n* = 6). (G) Western blot analysis of p16 and p21 protein levels in senescent VSMCs after HSP90α knockdown. (H) Senescence‐associated β‐galactosidase staining of senescent VSMCs after HSP90α knockdown. (I) Protein expression levels in senescent VSMCs after HSP90α knockdown. (J) Heatmap of expression changes of the top 10 proteins in senescent VSMCs after HSP90α knockdown. ^*^ *p* < 0.05; ^**^ *p* < 0.01. NS, non‐significant.

To characterize the role of HSP90α in senescent VSMCs, a senescent VSMC model was established using PA‐BSA (Figure 1A) [10]. VSMCs transitioned into a stable senescent state following treatment with PA‑BSA for 48 h and subsequent culture in PA‑BSA‑free medium for another 48 h, as evidenced by elevated SA‑β‑gal activity and upregulation of the cell cycle inhibitors p16 and p21 (Figure 1B,C). Upon entering senescence, VSMCs exhibited an increase in HSP90α levels (Figure 1D–F).

To determine whether the upregulated HSP90α sustains the survival of senescent VSMCs, an HSP90α knockdown was performed using siRNA (Figure S1). After HSP90α knockdown, the levels of senescence markers were significantly downregulated (Figure 1G,H). Further tandem mass tag‐based quantitative proteomics analyses revealed that HSP90α knockdown specifically activated the PERK/eIF2α/ATF4/CHOP pathway (Figure 1I,J), suggesting that high level of HSP90α inhibits ERS in senescent VSMCs. Together, these results suggest that HSP90α maintains senescent VSMC survival, potentially by suppressing ERS signaling.

### 3.2 Suppression of ERS Maintains Survival of Senescent VSMCs

**FIGURE 2:**
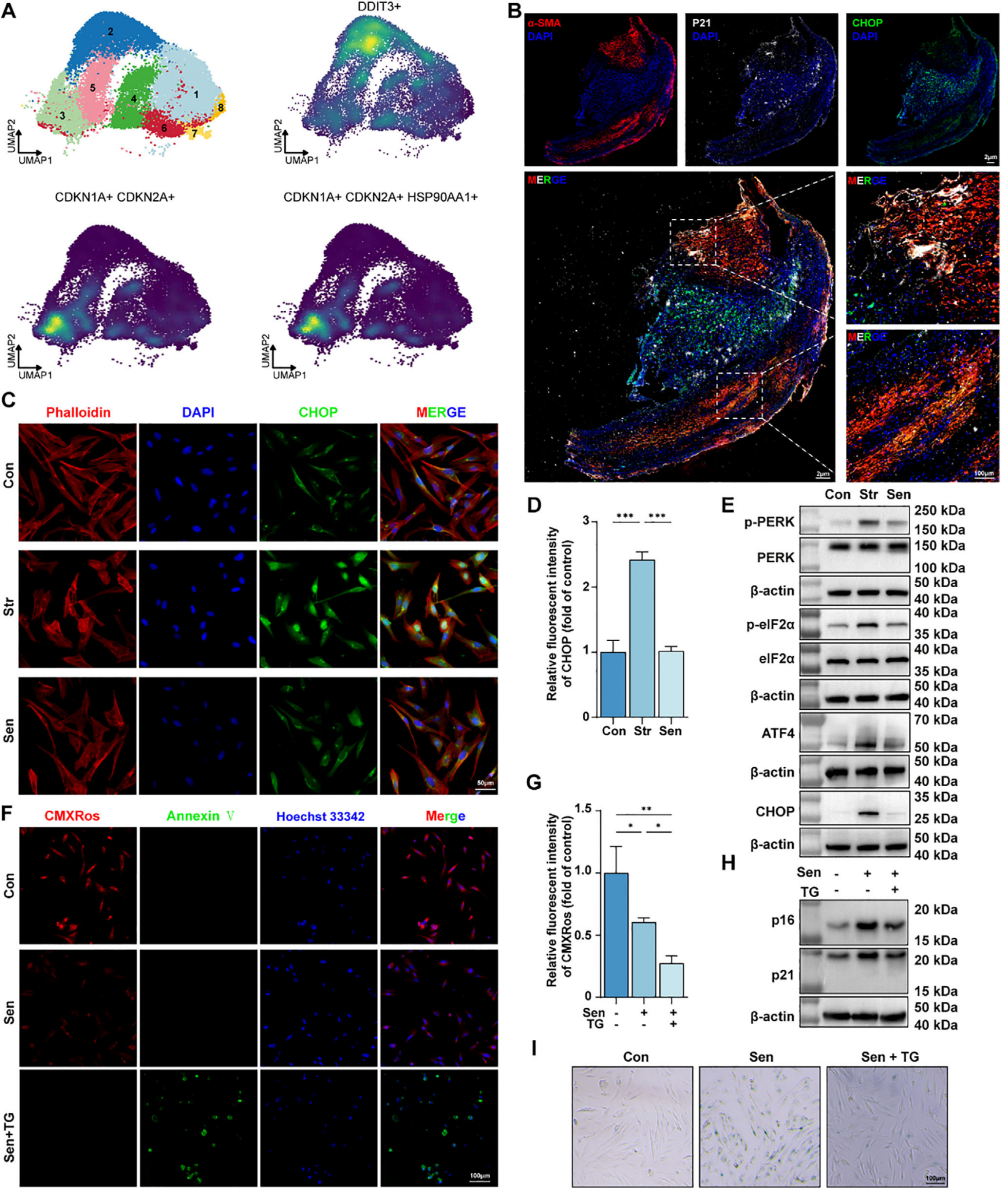
Suppression of *ERS* maintains senescent VSMC survival. (A) UMAP plot of the expression level distribution of TAGLN, CDKN1A, and DDIT3 genes in the cells from a coronary artery RNA‐seq dataset. (B) Immunofluorescence of the human atherosclerotic core and proximally adjacent region in the human carotid artery. (C) Representative immunofluorescence images of CHOP (green), phalloidin (red), and DAPI (blue)‐stained nuclei in stressed (Str) and senescent (Sen) VSMCs. (D) Quantification of CHOP (green) in stressed and senescent VSMCs (n = 6). (E) Western blot analysis of components of the PERK signaling pathway in stressed and senescent VSMCs. (F) Mito‐Tracker Red CMXRos and Annexin V‐FITC staining of VSMCs after 0.1 µm thapsigargin (TG) treatment. (G) Quantification of CMXRos (red) in VSMCs after 0.1 µm TG treatment (*n* = 6). (H) Western blot analysis of p16 and p21 protein levels in VSMCs after 0.1 µm TG treatment. (I) Senescence‐associated β‐galactosidase staining of VSMCs after 0.1 µm TG treatment. ^*^ *p* < 0.05; ^**^ *p* < 0.01; ^***^ *p* < 0.001.

We next characterized ERS in senescent VSMCs within human carotid atherosclerotic plaques by integrating four public atherosclerotic scRNA‑seq datasets comprising 40 samples [16, 17, 18, 19] (Table S3). Cluster 3 was identified as senescent VSMCs based on co‐expression of p21 and p16. HSP90α is highly expressed in senescent VSMCs, but it does not co‐localize with DDIT3 (CHOP) in these cells (Figure 2A).

Immunofluorescence staining of human carotid plaques corroborated this finding, revealing heterogeneous spatial distribution of CHOP and p21 proteins within the VSMC population (Figure 2B). Consistent with in vivo observations, cultured senescent VSMCs exhibited a suppressed PERK/eIF2α/ATF4/CHOP signaling pathway upon entering senescence (Figure 2C,E), but not the ATF6 or IRE1 axes (Figure S2). These results indicate that the PERK signaling is specifically suppressed in senescent VSMCs.

We hypothesized that reactivation of the suppressed PERK pathway would induce apoptosis in senescent VSMCs. To test this, senescent VSMCs were treated with thapsigargin, a specific activator of ERS. Treatment with 0.1 µm thapsigargin significantly reduced mitochondrial membrane potential while increasing the percentage of Annexin V‑FITC^+^ VSMCs (Figure S3 and Figure 2F,G). Thapsigargin treatment decreased the levels of p16, p21, and β‑galactosidase activity (Figure 2H,I). These findings suggest that reactivation of ERS triggers apoptosis in senescent VSMCs.

Given that PERK activation is considered to promote VSMC phenotypic switching [29], we next established a PERK knockdown VSMC model (Figure S1). PERK knockdown decreased p16 and p21 levels while restoring α‑SMA, SMMHC, and collagen I (Figure S4), all of which are recognized as markers of VSMC contractile phenotype and plaque stability [30]. However, neither PA‑BSA nor PERK knockdown affected the expression of the macrophage marker CD68 (Figure S4).

### 3.3 HSP90α Scaffolds the AKT‐PERK Complex to Inhibit PERK Activation in Senescent VSMCs

**FIGURE 3:**
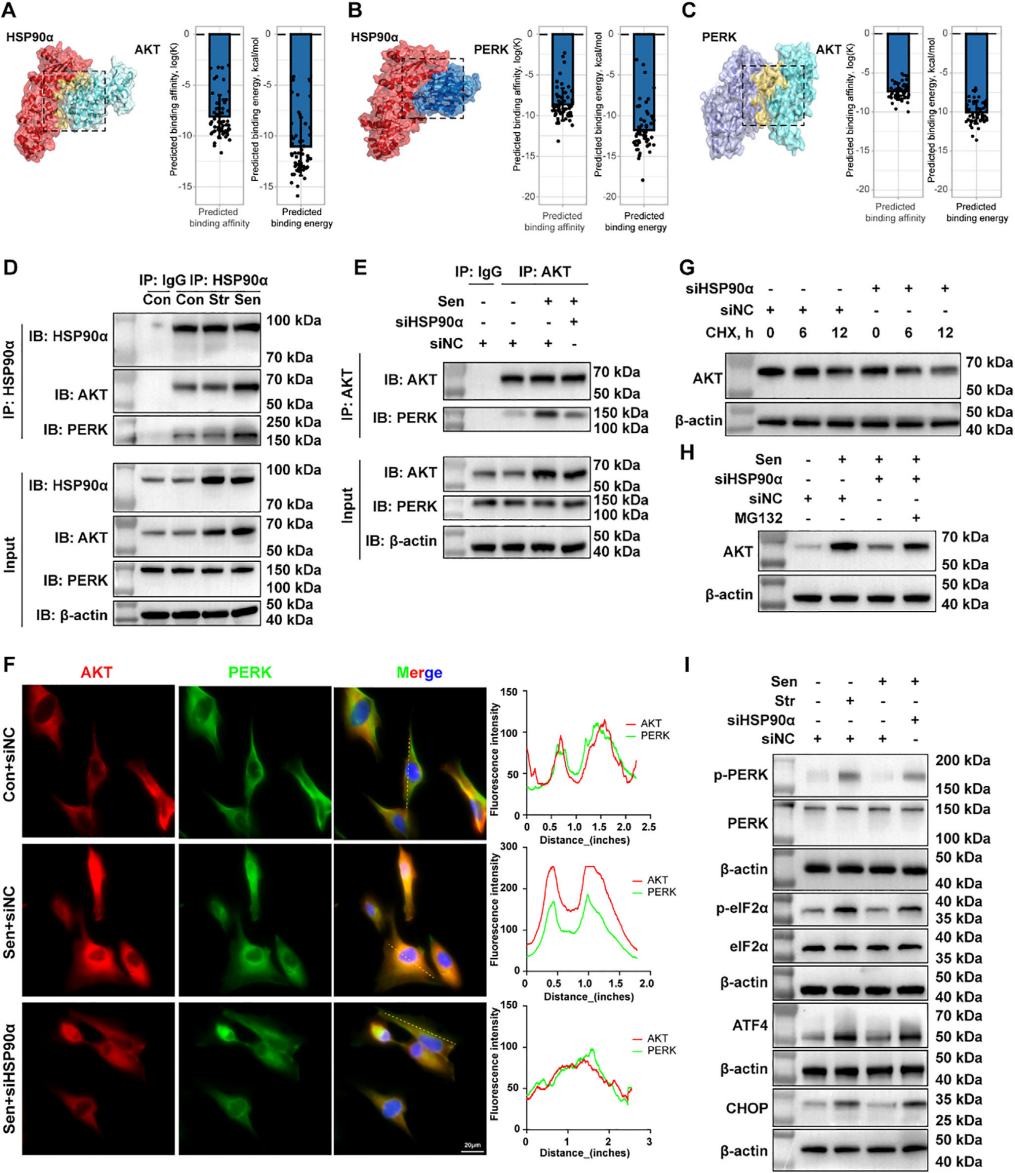
HSP90α scaffolds the AKT‐PERK complex to inhibit ERS activation in senescent VSMCs. (A–C) Results of in silico analyses of protein‐protein docking between HSP90α and AKT (A), HSP90α and PERK (B), and AKT and PERK (C). (D) Co‐immunoprecipitation assays of binding among HSP90α, AKT, and PERK. (E) Co‐immunoprecipitation assay of the binding between AKT and PERK in VSMCs after HSP90α knockdown. (F) Representative immunofluorescence images of AKT (red), PERK (green), and DAPI‐stained nuclei (blue) in HSP90α‐knockdown VSMCs. (G) Cycloheximide (CHX)‐based assay of AKT protein stability in senescent HSP90α‐knockdown VSMCs. (H) Western blot analysis of AKT levels in senescent HSP90α‐knockdown VSMCs treated with the proteasome inhibitor MG132. (I) Western blot analysis of the PERK signaling pathway in HSP90α‐knockdown VSMCs.

Previous studies have shown that AKT binds to the autophosphorylation site of PERK, thereby inhibiting PERK activation [31]. HSP90α acts as a scaffold to stabilize protein‐protein interactions [32]. Based on these established interactions, we investigated the role of HSP90α in AKT‐PERK interaction. Molecular docking assays identified pairwise interactions between HSP90α and AKT, between HSP90α and PERK, and between PERK and AKT (Figure 3A–C). Co‐immunoprecipitation assays further confirmed that their binding was specifically enhanced in senescent VSMCs (Figure 3D). To map the domain of HSP90AA1 involved in its interactions with AKT and PERK, we expressed the N‐terminal, C‐terminal, and middle domain of HSP90AA1 and showed that the middle region of HSP90AA1 (amino acid residues 284–620) was sufficient to bind to AKT and PERK (Figure S5). The PERK/AKT interaction was reduced upon HSP90α knockdown (Figure 3E,F).

To determine the role of HSP90α, we assessed AKT degradation and PERK activation in HSP90α‐knockdown senescent VSMCs. HSP90α knockdown accelerated AKT degradation (Figure 3G,H) and promoted the activation of PERK/eIF2α/ATF4/CHOP signaling (Figure 3I). Collectively, HSP90α levels are elevated in senescent VSMCs, where it maintains the AKT‐PERK interaction and thereby suppresses the activation of PERK signaling.

### 3.4 Reactivation of PERK Triggers Apoptosis of Senescent VSMCs via ER‐Mitochondrial Calcium Transfer

**FIGURE 4:**
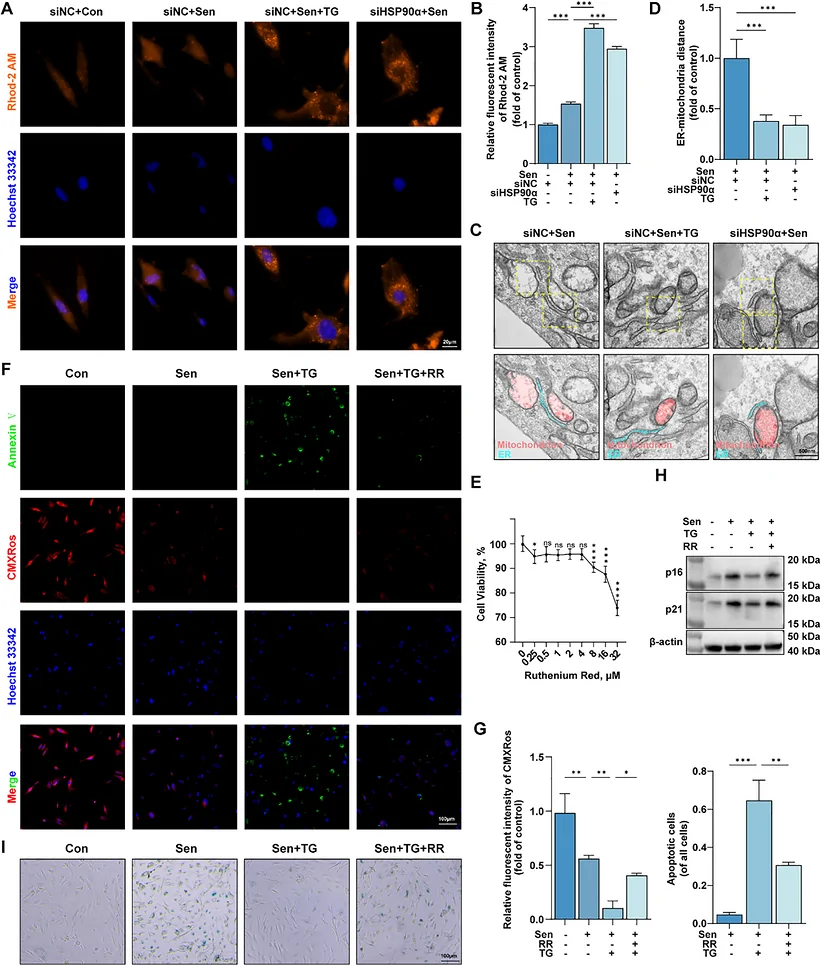
Activation of PERK induces apoptosis in senescent VSMCs via ER‐mitochondrial calcium transfer. (A) Representative images of mitochondrial calcium labeling (Rhod‐2 AM) in senescent VSMCs after treatment with 0.1 µm thapsigargin (TG) or HSP90α knockdown. (B) Quantification of mitochondrial calcium levels from images like those shown in panel (A) (n = 6). (C) Representative transmission electron microscopy images of mitochondria‐associated ER membranes in senescent VSMCs after treatment with 0.1 µm thapsigargin or HSP90α knockdown. (D) Quantification of ER‐mitochondria contact distance from panel (C) (*n* = 6). (E) Dose‐response curves of ruthenium red‐treated senescent VSMCs. (F) Mito‐Tracker Red CMXRos and Annexin V‐FITC staining of senescent VSMCs pretreated with 4 µm ruthenium red followed by 0.1 µm thapsigargin treatment. (G) Quantification of CMXRos (red) and Annexin V‐FITC (green) from panel (F) (*n* = 6). (H) Western blot analysis of p16 and p21 protein levels in senescent VSMCs pretreated with 4 µm ruthenium red followed by 0.1 µm thapsigargin treatment. (I) Senescence‐associated β‐galactosidase staining of senescent VSMCs pretreated with 4 µm ruthenium red followed by 0.1 µm thapsigargin treatment. ^*^ *p* < 0.05; ^**^ *p* < 0.01; ^***^ *p* < 0.001. Sen, senescent. RR, ruthenium red.

PERK signaling has been shown to trigger mitochondrial calcium overload [33, 34]. Accordingly, we assessed mitochondrial calcium levels in senescent VSMCs. Mitochondrial calcium labeling with Rhod‐2 AM demonstrated that HSP90α knockdown or thapsigargin treatment elevated mitochondrial calcium levels in senescent VSMCs (Figure 4A,B), which was further supported by increased ER‐mitochondrial contact area indicated by TEM (Figure 4C,D) and organelle‐specific probe labeling (Figure S6).

We therefore hypothesized that mitochondrial calcium uptake elicits apoptosis in senescent VSMCs. To test this hypothesis, senescent VSMCs were treated with ruthenium red, a specific inhibitor of mitochondrial calcium uptake. Pretreatment with 4 µm ruthenium red significantly abrogated thapsigargin‐induced apoptosis and the subsequent reduction in senescent cell burden (Figure 4E–I). These findings indicate that HSP90α inhibition and subsequent PERK reactivation induce apoptosis in senescent VSMCs by promoting mitochondrial calcium uptake.

### 3.5 Identification of SAA as an HSP90α Inhibitor That Induces Apoptosis of Senescent VSMCs

**FIGURE 5:**
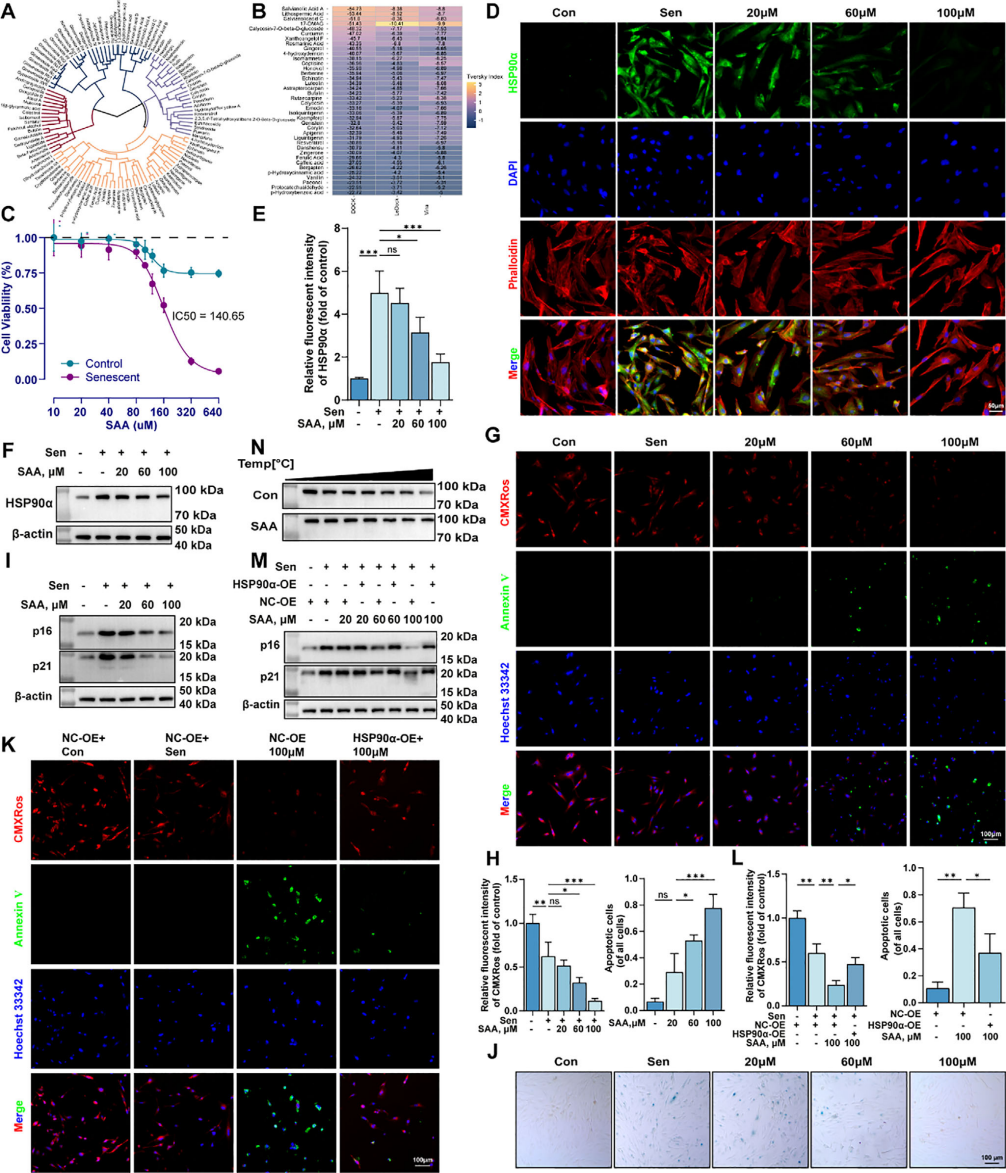
Identification of salvianolic acid A (SAA) as an HSP90α inhibitor that induces apoptosis of senescent VSMCs. (A) Hierarchical clustering analysis of 112 compounds based on FCFP6 circular fingerprints. (B) Target screening results based on compound profiles. (C) Dose‐response curves of SAA‐treated senescent or non‐senescent VSMCs. (D) Representative immunofluorescence images of HSP90α (green), phalloidin (red), and DAPI (blue)‐stained nuclei in VSMCs after SAA treatment. (E) Quantification of HSP90α (green) in VSMCs after SAA treatment (n = 6). (F) Western blot analysis of HSP90α protein levels. (G) Mito‐Tracker Red CMXRos and Annexin V‐FITC staining. (H) Quantification of CMXRos (red) and Annexin V‐FITC (green) (*n* = 6). (I) Western blot analysis of p16 and p21 protein levels. (J) Senescence‐associated β‐galactosidase staining. (K) Mito‐Tracker Red CMXRos and Annexin V‐FITC staining of VSMCs after SAA treatment following HSP90α overexpression. (L) Quantification of CMXRos (red) and Annexin V‐FITC (green) in VSMCs after SAA treatment following HSP90α overexpression (*n* = 6). (M) Western blot analysis of p16 and p21 protein levels in VSMCs after SAA treatment following HSP90α overexpression. (N) Cellular thermal shift assay results. ^*^ *p* < 0.05; ^**^ *p* < 0.01; ^***^ *p* < 0.001. Sen, senescent.

To identify natural compounds with dual anti‐tumor and anti‐atherosclerotic activity, we employed a standardized search strategy and established a dataset of 112 natural products (Figure 5A). Molecular docking was then performed between each of these 112 compounds and HSP90α to screen for candidates with high binding affinity to HSP90α. The reliability of the docking‐derived affinity scores was validated using 17‐DMAG, a well‐characterized HSP90α inhibitor. This analysis identified SAA as the top‐ranked compound, with a docking score comparable to that of 17‐DMAG (Figure 5B).

Senescent VSMCs were then treated with SAA at 20, 60, and 100 µm (Figure 5C). HSP90α was significantly inhibited in senescent VSMCs after SAA treatment (Figure 5D–F). SAA treatment markedly reduced mitochondrial membrane potential and increased Annexin V‐FITC^+^ cells (Figure 5G,H), and decreased the expression of p16 and p21, as well as β‑galactosidase activity (Figure 5I,J). However, HSP90α overexpression reversed SAA‑induced apoptosis in senescent VSMCs (Figure S1 and Figure 5K–M).

According to previous studies, IL‑6 and IL‑1α are representative SASP factors secreted by senescent VSMCs [10, 35]. The levels of IL‐1α and IL‐6 were elevated in the culture supernatants of senescent VSMCs, whereas SAA treatment suppressed their secretion (Figure S7). SAA treatment restored the expression of SMMHC, α‐SMA, and collagen I, but CD68 remained unaffected (Figure S8). Our study showed that SAA did not induce apoptosis in normal VSMCs (Figure S9).

To investigate whether SAA directly binds to HSP90α, we performed a cellular thermal shift assay and a biotin pull‑down assay. The cellular thermal shift assay results indicated that SAA enhances the thermal stability of HSP90α (Figure 5N). Biotin pull‐down analysis using biotin‐conjugated SAA demonstrated that SAA directly precipitated endogenous HSP90α (Figure S10).

### 3.6 SAA Reactivates PERK Signaling by Disrupting the HSP90α‐AKT‐PERK Ternary Complex

**FIGURE 6:**
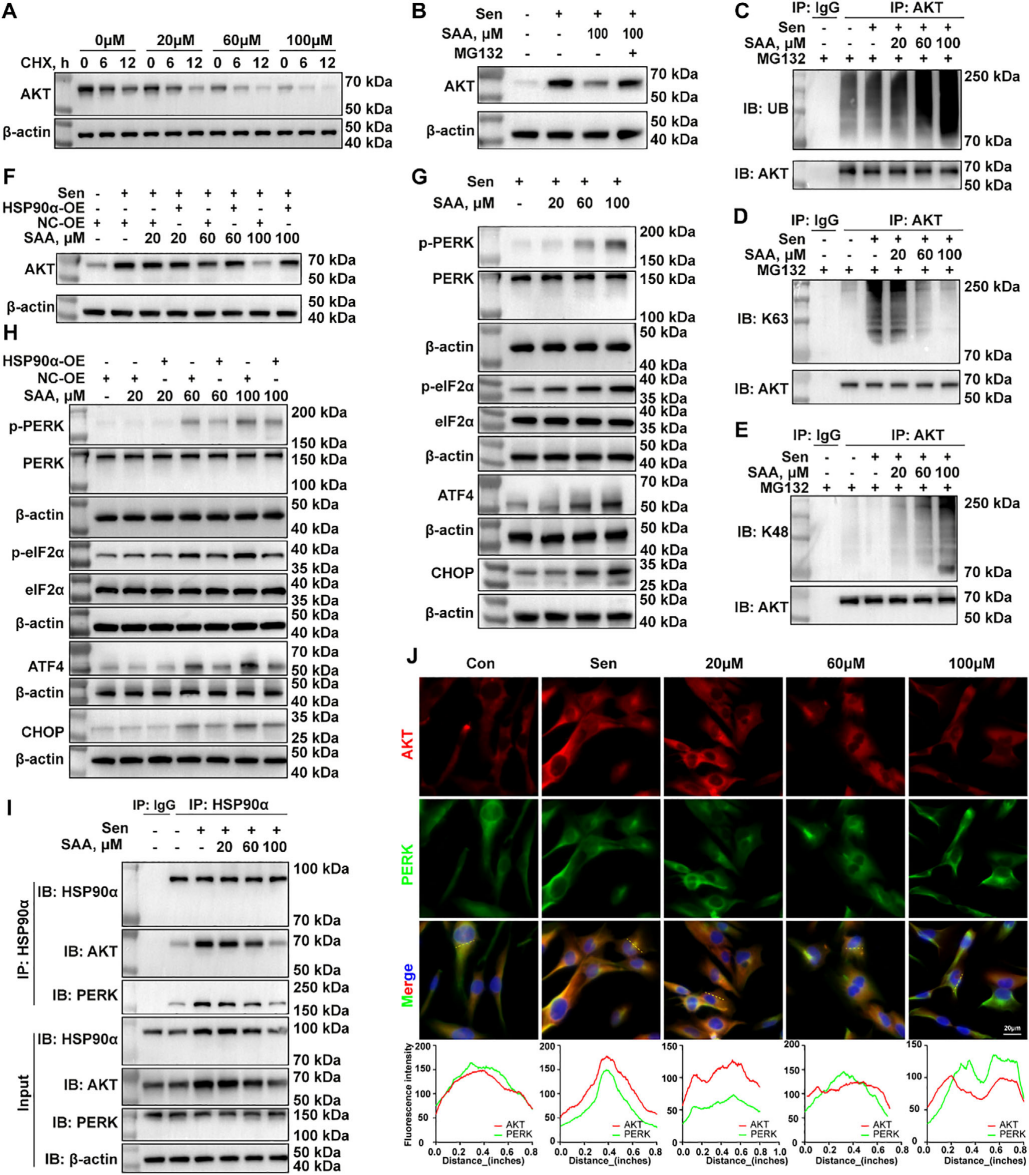
SAA reactivates ERS by disrupting the HSP90α‐AKT‐PERK ternary complex. (A) Cycloheximide‐based assay of AKT protein stability in senescent VSMCs after SAA treatment. (B) Western blot analysis of AKT levels in VSMCs after SAA treatment under the administration of the proteasome inhibitor MG132. (C) Western blot analysis of total ubiquitination levels of AKT in VSMCs after SAA treatment. (D) Western blot analysis of K63‐linked ubiquitination levels of AKT in VSMCs after SAA treatment. (E) Western blot analysis of K48‐linked ubiquitination levels of AKT in VSMCs after SAA treatment. (F) Western blot analysis of AKT protein levels in VSMCs after SAA treatment following HSP90α overexpression. (G,H) Western blot analysis of PERK signaling pathway components in senescent VSMCs without (G) or with (H) HSP90α overexpression after SAA treatment. (I) Co‐immunoprecipitation assay of the binding among HSP90α, AKT, and PERK in VSMCs after SAA treatment. (J) Representative immunofluorescence images of AKT (red), PERK (green), and DAPI‐stained nuclei (blue) in VSMCs after SAA treatment. Sen, senescent.

To investigate the effects of SAA on the AKT‐PERK complex, we examined AKT degradation in senescent VSMCs following SAA treatment. SAA accelerated AKT degradation in a dose‐dependent manner, and pretreatment with MG132 significantly inhibited AKT degradation (Figure 6A,B). This observation led us to hypothesize that SAA promotes AKT degradation via the ubiquitin‐proteasome pathway. Indeed, SAA was found to increase the total ubiquitination of AKT in senescent VSMCs (Figure 6C).

To determine whether SAA preferentially enhances K48‐ or K63‐linked AKT ubiquitination, we analyzed the specific ubiquitin chain linkages on AKT. Senescent VSMCs exhibited strong K63‐linked ubiquitination of AKT (Figure 6D), whereas K48‐linked ubiquitination was unaffected (Figure 6E). However, SAA treatment led to enhanced K48‐linked ubiquitination (Figure 6E) but reduced K63‐linked ubiquitination of AKT (Figure 6D). HSP90α overexpression significantly rescued the SAA‐induced reduction in AKT protein levels (Figure 6F). These results indicate that SAA promotes K48‐linked AKT ubiquitination by inhibiting HSP90α, leading to a subsequent decrease in AKT protein.

We next investigated PERK signaling in SAA‑treated senescent VSMCs. SAA markedly activated PERK/eIF2α/ATF4/CHOP signaling (Figure 6G), whereas HSP90α overexpression reversed this effect (Figure 6H). Given that PERK signaling activation in contractile VSMCs is established to promote atherogenesis, we examined whether SAA activates the PERK signaling pathway specifically in senescent VSMCs. Our results showed that SAA did not induce PERK signaling activation in normal VSMCs (Figure S11), validating that PERK is not a direct target of SAA.

Finally, the impact of SAA on the HSP90α‐AKT‐PERK ternary complex in senescent VSMCs was assessed. The co‐immunoprecipitation results showed that SAA diminished the intermolecular interactions among HSP90α, AKT, and PERK (Figure 6I,J). The cross‐linking using the protein cross‐linker DSS showed that a distinct 340 kDa band corresponding to the combined molecular weights of HSP90α, AKT, and PERK (Figure S12). SAA treatment reduced the intensity of this 340‐kDa complex band, further supporting our conclusion. Collectively, our findings are consistent with a model in which SAA inhibits the scaffolding function of HSP90α for AKT‐PERK complex, thereby promoting degradation of AKT and activation of the PERK signaling.

### 3.7 SAA Drives Apoptosis in Senescent VSMCs via PERK Activation and ER‐Mitochondrial Calcium Transfer

**FIGURE 7:**
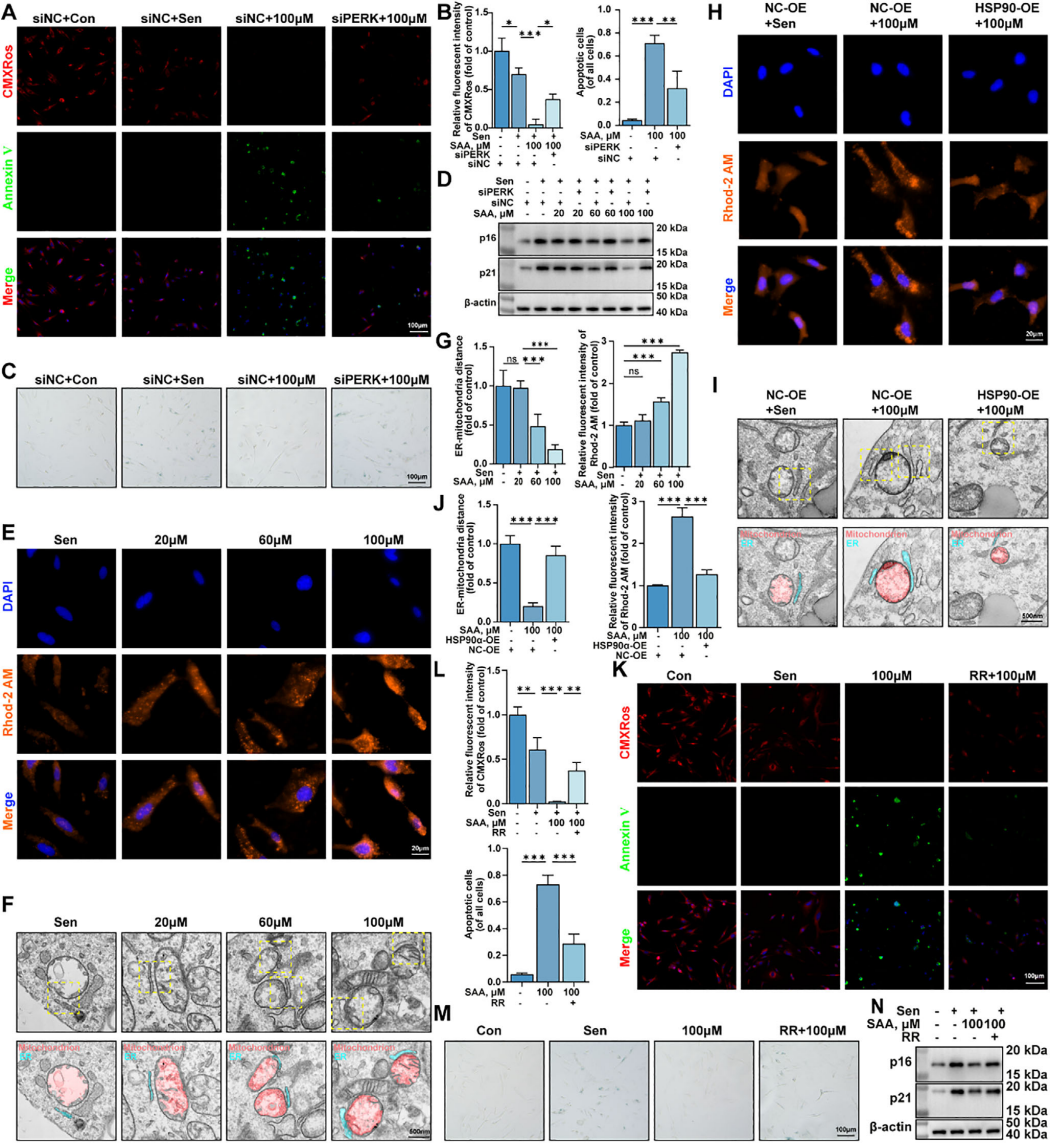
SAA drives apoptosis in senescent VSMCs via PERK activation and ER‐mitochondrial calcium transfer. (A) Mito‐Tracker Red CMXRos and Annexin V‐FITC staining of VSMCs after SAA treatment following PERK knockdown. (B) Quantification of staining of images as in (A) (n = 6). (C) Senescence‐associated β‐galactosidase staining of VSMCs after SAA treatment following PERK knockdown. (D) Western blot analysis of p16 and p21 protein levels in VSMCs after SAA treatment following PERK knockdown. (E) Representative images of mitochondrial calcium labeled by Rhod‐2 AM in senescent VSMCs after SAA treatment. (F) Representative transmission electron microscopy images of mitochondria‐associated ER membranes in senescent VSMCs after SAA treatment. (G) Quantification of mitochondrial calcium levels and ER‐mitochondria contact distance from images as shown in (E, F) (n = 6). (H) Representative images of mitochondrial calcium labeled by Rhod‐2 AM in senescent VSMCs after SAA treatment following HSP90α overexpression. (I) Representative transmission electron microscopy images of mitochondria‐associated ER membranes in senescent VSMCs after SAA treatment following HSP90α overexpression. (J) Quantification of mitochondrial calcium levels and ER‐mitochondria contact distance from images as shown in (H, I) (n = 6). (K) Mito‐Tracker Red CMXRos and Annexin V‐FITC staining of VSMCs pretreated with 4 µm ruthenium red followed by SAA treatment. (L) Quantification of labelling as shown in (K) (*n* = 6). (M) Senescence‐associated β‐galactosidase staining of VSMCs pretreated with 4 µm ruthenium red followed by SAA treatment. (N) Western blot analysis of p16 and p21 protein levels in VSMCs pretreated with 4 µm ruthenium red followed by SAA treatment. ^*^ *p* < 0.05; ^**^ *p* < 0.01; ^***^ *p* < 0.001. Sen, senescent. RR, ruthenium red.

To investigate whether the induction of apoptosis by SAA in senescent VSMCs was mediated by the PERK signaling, a PERK‐knockdown senescent VSMC model was then established. PERK knockdown significantly reversed the SAA‐induced reduction in senescence markers and rescued apoptosis (Figure 7A–D). SAA enhanced mitochondrial calcium uptake and reduced ER‐mitochondrial contact distance (Figure 7E–G and Figure S13), whereas HSP90α overexpression counteracted the effects of SAA (Figure 7H–J and Figure S14).

We therefore hypothesized that SAA‐induced ER‐mitochondrial calcium transfer mediates apoptosis. To test this, ruthenium red was administered to senescent VSMCs before SAA treatment, which significantly attenuated the pro‑apoptotic effect of SAA (Figure 7K–N). The MCU is a critical channel for ER‑mitochondria calcium flux [36]. We then conducted loss‑of‑function experiments using MCU‑specific siRNA (Figure S1) and observed that MCU knockdown suppressed SAA‑induced apoptosis in VSMCs, providing further support for our results (Figure S15). These data indicate that SAA induces apoptosis in senescent VSMCs via ERS activation and subsequent ER‐mitochondrial calcium transfer.

### 3.8 SAA Attenuates Atherosclerosis and Stabilizes Plaques by Reducing the Senescent VSMC Burden

**FIGURE 8:**
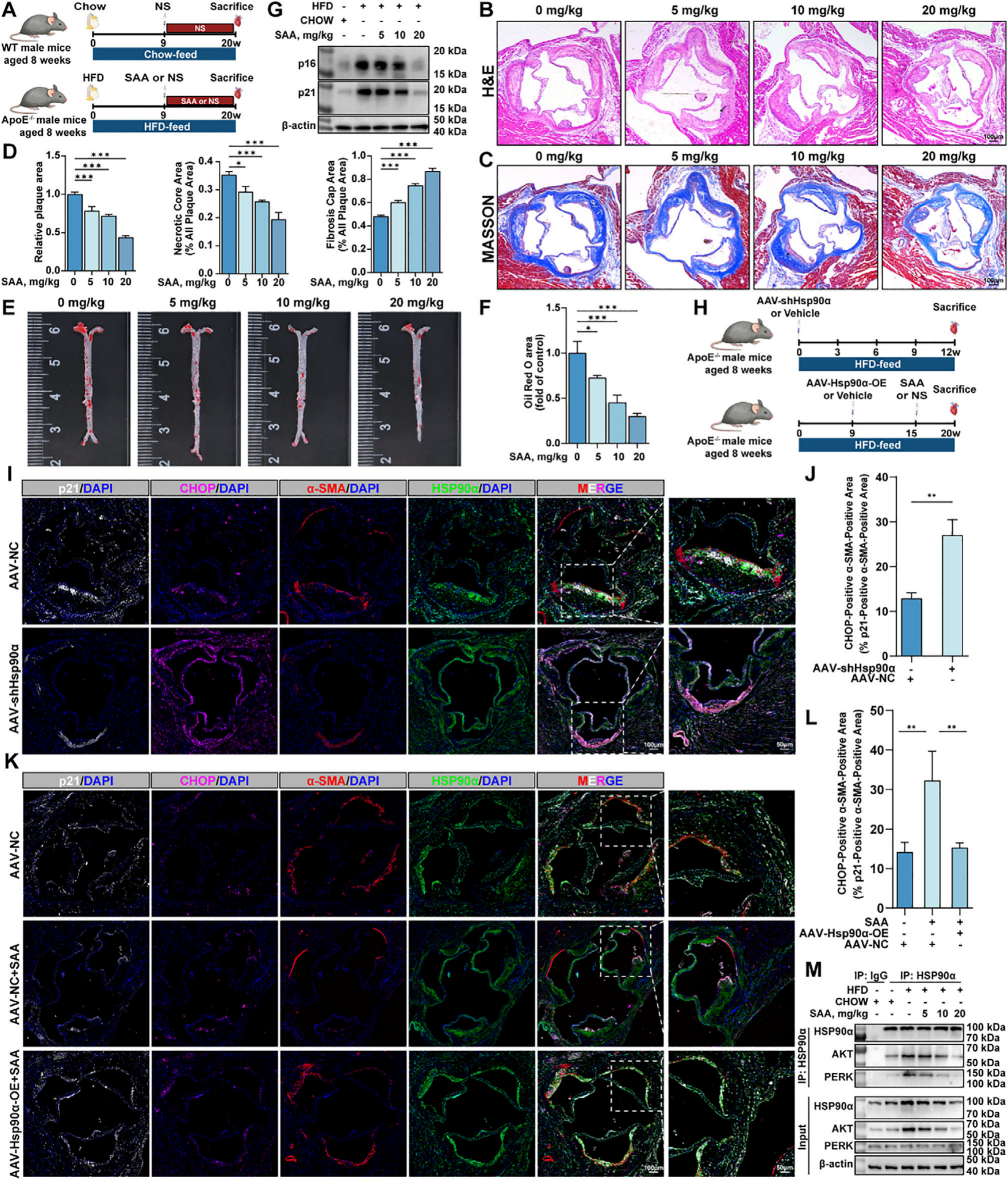
SAA attenuates atherosclerosis and stabilizes plaques by reducing the senescent VSMC burden. (A) Schematic diagram of the animal experimental design. (B) H&E and (C) Masson staining images in aortic root of mice after VSMCs treatment. (D) Quantification of plaque, fibrosis cap, and necrotic core area in aortic root of mice after VSMCs treatment (*n* = 6). (E) Oil Red O staining of mouse aortas. (F) Quantification of Oil Red O‐positive area from images as in (E) (*n* = 6). (G) Western blot analysis of p16 and p21 protein levels in mouse aortas. (H) Schematic diagram of the animal experimental design. (I) Representative immunofluorescence images of p21 (white), CHOP (purple), α‐SMA (red), HSP90α (green), and nuclei stained with DAPI (blue) in aortic root of ApoE^−/−^ mice after VSMCs‐specific HSP90α knockdown. (J) Quantification of immunofluorescence as in panel (I) (*n* = 6). (K) Representative immunofluorescence images of p21 (white), CHOP (purple), α‐SMA (red), HSP90α (green), and nuclei stained with DAPI (blue) in aortic root of ApoE^−/−^ mice after VSMCs‐specific HSP90α overexpression. (L) Quantification of immunofluorescence as in panel (K) (*n* = 6). (M) Co‐immunoprecipitation assay of the interactions among HSP90α, AKT, and PERK in mouse aortas after in vivo SAA treatment. ^*^ *p* < 0.05; ^**^ *p* < 0.01; ^***^ *p* < 0.001. NS, normal saline; HFD, High‐Fat Diet.

To investigate the in vivo effects of SAA on atherosclerotic progression, ApoE^−/−^ mice were fed a HFD for 20 weeks and treated intraperitoneally with SAA or vehicle from week 9 for 12 weeks (Figure 8A). SAA markedly attenuated the pathological progression of atherosclerosis, as evidenced by decreased plaque area, reduced necrotic core size, and increased fibrous cap thickness (Figure 8B–D). SAA treatment markedly decreased lipid accumulation in the aorta (Figure 8E,F). IL‐6 is a hallmark SASP factor secreted by senescent cells. SAA significantly reduced IL‐6 levels in the serum of atherosclerotic mice (Figure S16).

SAA markedly downregulated the expression of p21 and p16 in aorta (Figure 8G). Since our in vitro experiments showed that SAA activates PERK in an HSP90α‐dependent manner, we generated an Hsp90aa1 knockdown AAV to specifically knock down HSP90α expression in VSMCs of ApoE^−/−^ mice (Figure 8H). VSMC‐specific knockdown of HSP90α significantly increased the colocalization of p21 and CHOP in VSMCs (Figure 8I,J). In contrast, VSMC‐specific HSP90α overexpression markedly inhibited SAA‐induced p21‐CHOP colocalization (Figure 8K,L). HSP90α overexpression led to a rebound of p21^+^ VSMCs, whereas HSP90α knockdown and SAA treatment decreased their abundance (Figure S17).

Finally, we examined the effect of SAA on the HSP90α‐AKT‐PERK complex in ApoE^−/−^ mice and found that SAA reduced the intermolecular interaction among HSP90α, AKT, and PERK in the aorta (Figure 8M). Our findings indicate that SAA significantly reduces the senescent VSMC burden in atherosclerotic lesions by inhibiting HSP90α. Specifically, SAA treatment disrupts the HSP90α‐AKT‐PERK complex, activates PERK signaling in senescent VSMCs, and thereby induces apoptosis in these cells.

## 4 Discussion

Our study provides evidences that high expression of HSP90α and suppression of PERK signaling mediate resistance to apoptosis in senescent VSMCs in atherosclerosis. Our results also present the novel finding that the scaffolding function of HSP90α in mediating the AKT‐PERK interaction accounts for the PERK suppression. Through virtual screening, SAA was identified as a potential HSP90α inhibitor that selectively induced apoptosis in senescent VSMCs. In ApoE^−^/^−^ mice, SAA significantly reduces the senescent VSMC burden and alleviates the progression of atherosclerosis.

HSP90α is a critical chaperone molecule that facilitates survival mechanisms in tumor cells [37]. Previous studies have demonstrated that HSP90 is upregulated in inflammatory regions and thin‐cap plaques within human atherosclerotic lesions and that it decreases plaque stability [38]. Treatment with known HSP90 inhibitors has been reported to attenuate atherosclerotic progression and stabilize plaques [38, 39, 40]. In patients with diabetic atherosclerosis, extracellular secretion of HSP90α is significantly increased and in turn exacerbates inflammatory responses and vascular injury [41]. In our study, it was suggested that HSP90α maintains the viability of senescent VSMCs.

HSP90α is traditionally defined as a molecular chaperone that maintains intracellular homeostasis by facilitating the folding and conformational stability of proteins [42, 43]. Recent studies have demonstrated that HSP90 can form higher‐order oligomeric forms and remodel protein‐protein interaction networks, which is referred to as its protein scaffolding function [32]. In cancer, HSP90 oligomers induce dysfunction of protein‐protein interactions [44, 45]. In Alzheimer's disease, HSP90 oligomers modulate the interactions of proteins associated with synaptic plasticity [46]. In our study, we showed that HSP90α acts as a scaffold to maintain the AKT‐PERK interaction, thereby mediating the suppression of PERK signaling of senescent VSMCs.

ERS determines cell fate under stressful conditions [47, 48, 49]. PERK activation promotes aberrant phenotypic switching of VSMCs, including the transition toward senescence [29, 50, 51]. However, unlike contractile VSMCs, senescent VSMCs exhibit attenuated PERK activation in response to stress insults, a phenomenon also reported in senescent fibroblasts [52]. In our study, we found that PERK signaling, but not the ATF6 and IRE1 axes, is specifically suppressed in senescent VSMCs, which may be attributable to the distinct role of the PERK/eIF2α axis. Under stress insults, eIF2α is phosphorylated by PERK to induce the suppression of global protein translation, which is incompatible with the high metabolic activity of senescent cells [52, 53]. This dissociation of the unfolded protein response branches has also been reported in aged mice and aged humans [54, 55]. Another study has also shown that senescent cells need to sustain SASP secretion even under hypoxia and amino acid deprivation, which is achieved by acquiring reduced sensitivity to PERK signaling [56]. Taken together, PERK is gradually downregulated during the stress‐to‐senescence transition to meet the survival demands of senescent cells, which is more likely a consequence of an active adaptive strategy of these cells. PERK alteration did not affect macrophage‑like switching, possibly because PA stimulation was insufficient to fully mimic cholesterol loading, which is considered a key initiating factor for VSMC phenotypic switching [57].

Mitochondrial calcium dyshomeostasis is a key downstream signal of PERK activation [58]. The activation of PERK signaling reduces the physical distance between the ER and mitochondria, thereby promoting ER‑to‑mitochondria calcium transfer via the MCU channel [59]. Mitochondrial calcium overload leads to excessive mitochondrial fission and collapse of the mitochondrial membrane potential, thereby inducing apoptosis [60]. It has been reported that the capacity for mitochondrial calcium uptake progressively decreases during fibroblast senescence [61], which is consistent with our findings.

SAA, a natural product derived from *Salvia miltiorrhiza*, exhibits bioactivities against both tumors and atherosclerosis. Herein, we identified SAA as a senolytic agent that directly binds to HSP90α and specifically induces PERK‑dependent apoptosis in senescent VSMCs, while exerting no apoptotic effect on normal VSMCs. PERK activation exerts either pro‑apoptotic or pro‑adaptive effects depending on the context [62]. Our study showed that SAA triggers apoptosis in senescent VSMCs rather than cell survival, which is supported by the activation of the ATF4‑CHOP axis. Consistent with the in vitro findings, SAA activated CHOP signaling in senescent VSMCs within atherosclerotic plaques, reduced the number of p21^+^ VSMCs, and attenuated atherosclerotic lesion progression in vivo. SAA has also been reported to inhibit endothelial‑mesenchymal transition, thereby attenuating atherosclerosis [63]. Our study, on the other hand, for the first time provides evidence for the anti‑atherosclerotic effect of SAA from the perspective of senolysis. It is a promising approach to attenuate atherosclerosis and prolong lifespan, but it faces difficulties in clinical translation due to cardiotoxicity [64].

In previous high‐throughput screens of compound libraries, the HSP90 inhibitors geldanamycin, tanespimycin, and 17‐DMAG were identified as senolytics that specifically trigger apoptosis in senescent fibroblasts [13]. Traditional HSP90 inhibitors were originally developed for anticancer therapy, but their clinical translation has been hampered by severe toxicity, with no successful drug approvals to date [65]. Exploring phytochemical‐rich plant extracts as candidates for healthspan‐extending formulations is a highly promising direction. Standardized extracts of *Salvia haenkei* have been shown to prolong both lifespan and healthspan in naturally aged mice, as well as ameliorate stress‐induced senescence [66]. SAA, a bioactive component derived from Salvia species, exemplifies the potential of plant‐derived compounds for anti‐aging and cardiovascular protection [67].

Importantly, SAA is in a phase II clinical trial for the treatment of diabetic microangiopathy (CTR20210544) and in two phase I clinical trials for the treatment of angina pectoris and diabetes (CTR20181023 and NCT03908242). This indicates that SAA has favorable safety and clinical translational potential. Our current work supports the role of SAA in stabilizing vulnerable plaques. However, translation from mouse models to humans is highly complex, and whether SAA can reverse plaque progression warrants future well‐designed endpoint clinical trials and further mechanistic investigations.

## 5 Conclusion

This study identified a novel mechanism in which HSP90α promotes survival of senescent VSMCs by scaffolding AKT‐PERK interaction to suppress the activation of PERK signaling. From a traditional Chinese medicine library, this study identified SAA as a HSP90α inhibitor to reduce the senescent VSMC burden, thereby alleviating atherosclerosis in ApoE^−^/^−^ mice. Our findings provide novel mechanistic insights into apoptosis resistance in senescent VSMCs and offer new therapeutic targets and potential agents for slowing the progression of atherosclerosis.

## Author Contributions

**Xiuya Guan**: conceptualization, methodology, software, investigation, validation, formal analysis, visualization, writing – original draft. **Mengkai Lu**: conceptualization, methodology, software, investigation, validation. **Xinhai Cui**: conceptualization, investigation, methodology, software. **Dawei Zhang**: methodology, validation, formal analysis. **Lei Zhang**: methodology, validation, formal analysis. **Lin Lin**: methodology, validation, formal analysis. **Yuan Li**: methodology, validation, formal analysis. **Yuanlong Hu**: methodology, conceptualization, supervision, investigation, writing – review and editing. **Chao Li**: conceptualization, methodology, data curation, supervision, resources, project administration, funding acquisition, writing – review and editing.

## Funding

Our study was supported by National Nature Science Foundation of China (82374408), Shandong Provincial Natural Science Foundation (ZR2025QB42), Special Fund for Taishan Scholar Project (tsqn202408173), and China Postdoctoral Science Foundation (2025T181071 and 2024M751904).

## Ethics Statement

Human samples were obtained with written informed consent and approval from the Research Ethics Committee of Affiliated Hospital of Shandong University of Traditional Chinese Medicine (No. 2025‐034‐KY), which conforms to the principles outlined in the Declaration of Helsinki. All the animal procedures were approved by the Animal Care and Use Committee of the School of Life Science and Technology, Shandong University of Traditional Chinese Medicine (No. SDUTCM20241112001).

## Consent

Informed written consent was obtained prior to the collection of the samples.

## Conflicts of Interest

The authors declare no conflicts of interest.

## Supporting information




**Supporting File**: advs77891‐sup‐0001‐SuppMat.docx.

## Data Availability

The data that support the current study are available in the supplementary materials of this article.

## References

[1] J. Cheng , H. Huang , Y. Chen , and R. Wu , “Nanomedicine for Diagnosis and Treatment of Atherosclerosis,” Advanced Science 10, no. 36 (2023): 2304294, 10.1002/advs.202304294. PMC10754137 37897322

[2] Y. Liu , K. Lu , R. Zhang , et al., “Advancements in the Treatment of Atherosclerosis: From Conventional Therapies to Cutting‐Edge Innovations,” ACS pharmacology & translational science 7, no. 12 (2024): 3804–3826, 10.1021/acsptsci.4c00574. PMC11651175 39698263

[3] K. E. Kypreos , R. Bitzur , E. A. Karavia , E. Xepapadaki , G. Panayiotakopoulos , and C. Constantinou , “Pharmacological Management of Dyslipidemia in Atherosclerosis: Limitations, Challenges, and New Therapeutic Opportunities,” Angiology 70, no. 3 (2019): 197–209, 10.1177/0003319718779533. 29862840

[4] P. Chatterjee and K. A. Martin , “A Concept of “Athero‐Oncology”: Tumor‐Like Smooth Muscle Cells Drive Atherosclerosis,” Circulation 149, no. 24 (2024): 1899–1902, 10.1161/circulationaha.124.069446. PMC12051038 38857330

[5] A. Kaistha , S. Oc , A. M. Garrido , et al., “Premature Cell Senescence Promotes Vascular Smooth Muscle Cell Phenotypic Modulation and Resistance to Re‐Differentiation,” Cardiovascular Research 121, no. 9 (2025): 1448–1463, 10.1093/cvr/cvaf102. PMC12352304 40493738

[6] M. O. J. Grootaert , M. Moulis , L. Roth , et al., “Vascular Smooth Muscle Cell Death, Autophagy and Senescence in Atherosclerosis,” Cardiovascular Research 114, no. 4 (2018): 622–634, 10.1093/cvr/cvy007. 29360955

[7] J. Wang , A. K. Uryga , J. Reinhold , et al., “Vascular Smooth Muscle Cell Senescence Promotes Atherosclerosis and Features of Plaque Vulnerability,” Circulation 132, no. 20 (2015): 1909–1919, 10.1161/CIRCULATIONAHA.115.016457. 26416809

[8] C. Chi , D.‐J. Li , Y.‐J. Jiang , et al., “Vascular Smooth Muscle Cell Senescence and Age‐Related Diseases: State of the Art,” Biochimica et Biophysica Acta (BBA)—Molecular Basis of Disease 1865, no. 7 (2019): 1810–1821, 10.1016/j.bbadis.2018.08.015. 31109451

[9] M. Xu , T. Tchkonia , and J. L. Kirkland , “Perspective: Targeting the JAK/STAT Pathway to Fight Age‐Related Dysfunction,” Pharmacological Research 111 (2016): 152–154, 10.1016/j.phrs.2016.05.015. PMC5026572 27241018

[10] M. O. J. Grootaert , A. Finigan , N. L. Figg , A. K. Uryga , and M. R. Bennett , “SIRT6 Protects Smooth Muscle Cells from Senescence and Reduces Atherosclerosis,” Circulation Research 128, no. 4 (2021): 474–491, 10.1161/CIRCRESAHA.120.318353. PMC7899748 33353368

[11] X. Li , M. Chen , X. Chen , et al., “TRAP1 Drives Smooth Muscle Cell Senescence and Promotes Atherosclerosis via HDAC3‐Primed Histone H4 Lysine 12 Lactylation,” European Heart Journal 45, no. 39 (2024): 4219–4235, 10.1093/eurheartj/ehae379. PMC11481199 39088352

[12] V. J. A. Barthet and S. W. Lowe , “Killing Wisely: Precision Senolytics in the Age of Frailty,” Genes & Development 39, no. 15–16 (2025): 910–913, 10.1101/gad.353134.125. PMC12315863 40664503

[13] H. Fuhrmann‐Stroissnigg , Y. Y. Ling , J. Zhao , et al., “Identification of HSP90 Inhibitors as a Novel Class of Senolytics,” Nature Communications 8, no. 1 (2017): 422, 10.1038/s41467-017-00314-z. PMC5583353 28871086

[14] J. Liu , D. Geremew , L. Y. Sandeman , et al., “Development of a Novel Murine Model of In‐Stent Neoatherosclerosis,” Journal of the American Heart Association 14, no. 22 (2025): 041260, 10.1161/JAHA.125.041260. PMC12887238 41220139

[15] A. Daugherty , A. R. Tall , M. J. A. P. Daemen , et al., “Recommendation on Design, Execution, and Reporting of Animal Atherosclerosis Studies: A Scientific Statement From the American Heart Association,” Circulation Research 121, no. 6 (2017): e53–e79, 10.1161/RES.0000000000000169. 28729353

[16] T. Alsaigh , D. Evans , D. Frankel , and A. Torkamani , “Decoding the Transcriptome of Calcified Atherosclerotic Plaque at Single‐Cell Resolution,” Communications Biology 5, no. 1 (2022): 1084, 10.1038/s42003-022-04056-7. PMC9556750 36224302

[17] A. C. Bashore , H. Yan , C. Xue , et al., “High‐Dimensional Single‐Cell Multimodal Landscape of Human Carotid Atherosclerosis,” Arteriosclerosis, Thrombosis, and Vascular Biology 44, no. 4 (2024): 930–945, 10.1161/ATVBAHA.123.320524. PMC10978277 38385291

[18] H. Pan , C. Xue , B. J. Auerbach , et al., “Single‐Cell Genomics Reveals a Novel Cell State during Smooth Muscle Cell Phenotypic Switching and Potential Therapeutic Targets for Atherosclerosis in Mouse and Human,” Circulation 142, no. 21 (2020): 2060–2075, 10.1161/CIRCULATIONAHA.120.048378. PMC8104264 32962412

[19] T. P. Fidler , A. Dunbar , E. Kim , et al., “Suppression of IL‐1β Promotes Beneficial Accumulation of Fibroblast‐Like Cells in Atherosclerotic Plaques in Clonal Hematopoiesis,” Nature Cardiovascular Research 3, no. 1 (2024): 60–75, 10.1038/s44161-023-00405-9. PMC10868728 38362011

[20] I. Korsunsky , N. Millard , J. Fan , et al., “Fast, Sensitive and Accurate Integration of Single‐Cell Data With Harmony,” Nature Methods 16, no. 12 (2019): 1289–1296, 10.1038/s41592-019-0619-0. PMC6884693 31740819

[21] J. Taminau , G. Thijs , and H. De Winter , “Pharao: Pharmacophore Alignment and Optimization,” Journal of Molecular Graphics and Modelling 27, no. 2 (2008): 161–169, 10.1016/j.jmgm.2008.04.003. 18485770

[22] A. F. A. Moumbock , J. Li , H. T. T. Tran , et al., “ePharmaLib: A Versatile Library of E‐Pharmacophores to Address Small‐Molecule (Poly‐)Pharmacology,” Journal of Chemical Information and Modeling 61, no. 7 (2021): 3659–3666, 10.1021/acs.jcim.1c00135. 34236848

[23] O. Trott and A. J. Olson , “AutoDock Vina: Improving the Speed and Accuracy of Docking With a New Scoring Function, Efficient Optimization, and Multithreading,” Journal of Computational Chemistry 31, no. 2 (2010): 455–461, 10.1002/jcc.21334. PMC3041641 19499576

[24] N. Zhang and H. Zhao , “Enriching Screening Libraries With Bioactive Fragment Space,” Bioorganic & Medicinal Chemistry Letters 26, no. 15 (2016): 3594–3597, 10.1016/j.bmcl.2016.06.013. 27311891

[25] J. M. Jez , J. C.‐H. Chen , G. Rastelli , R. M. Stroud , and D. V. Santi , “Crystal Structure and Molecular Modeling of 17‐DMAG in Complex With Human Hsp90,” Chemistry & Biology 10, no. 4 (2003): 361–368, 10.1016/s1074-5521(03)00075-9. 12725864

[26] R. Kolde , S. Laur , P. Adler , and J. Vilo , “Robust Rank Aggregation for Gene List Integration and Meta‐Analysis,” Bioinformatics 28, no. 4 (2012): 573–580, 10.1093/bioinformatics/btr709. PMC3278763 22247279

[27] Y. Yan , H. Tao , J. He , and S.‐Y. Huang , “The HDOCK Server for Integrated Protein–Protein Docking,” Nature Protocols 15, no. 5 (2020): 1829–1852, 10.1038/s41596-020-0312-x. 32269383

[28] Y. X. Yang , J. Y. Huang , P. Wang , and B. T. Zhu , “AREA‐AFFINITY: A Web Server for Machine Learning‐Based Prediction of Protein–Protein and Antibody–Protein Antigen Binding Affinities,” Journal of Chemical Information and Modeling 63, no. 11 (2023): 3230–3237, 10.1021/acs.jcim.2c01499. PMC10268951 37235532

[29] A. Chattopadhyay , C. S. Kwartler , K. Kaw , et al., “Cholesterol‐Induced Phenotypic Modulation of Smooth Muscle Cells to Macrophage/Fibroblast–Like Cells Is Driven by an Unfolded Protein Response,” Arteriosclerosis, Thrombosis, and Vascular Biology 41, no. 1 (2021): 302–316, 10.1161/ATVBAHA.120.315164. PMC7752246 33028096

[30] M. Zhai , Z. Lei , Y. Shi , et al., “TEAD1‐Mediated Trans‐Differentiation of Vascular Smooth Muscle Cells Into Fibroblast‐Like Cells Contributes to the Stabilization and Repair of Disrupted Atherosclerotic Plaques,” Advanced Science 12, no. 5 (2025): 2407408, 10.1002/advs.202407408. PMC11791998 39665254

[31] Z. Mounir , J. L. Krishnamoorthy , S. Wang , et al., “Akt Determines Cell Fate Through Inhibition of the PERK‐eIF2α Phosphorylation Pathway,” Science Signaling 4, no. 192 (2011): ra62, 10.1126/scisignal.2001630. PMC3752779 21954288

[32] G. Chiosis , C. S. Digwal , J. B. Trepel , and L. Neckers , “Structural and Functional Complexity of HSP90 in Cellular Homeostasis and Disease,” Nature Reviews Molecular Cell Biology 24, no. 11 (2023): 797–815, 10.1038/s41580-023-00640-9. PMC10592246 37524848

[33] N. Wang , C. Wang , H. Zhao , et al., “The MAMs Structure and Its Role in Cell Death,” Cells 10, no. 3 (2021): 657, 10.3390/cells10030657. PMC7999768 33809551

[34] S. Martucciello , M. Masullo , A. Cerulli , and S. Piacente , “Natural Products Targeting ER Stress, and the Functional Link to Mitochondria,” International Journal of Molecular Sciences 21, no. 6 (2020): 1905, 10.3390/ijms21061905. PMC7139827 32168739

[35] S. E. Gardner , M. Humphry , M. R. Bennett , and M. C. H. Clarke , “Senescent Vascular Smooth Muscle Cells Drive Inflammation Through an Interleukin‐1α–Dependent Senescence‐Associated Secretory Phenotype,” Arteriosclerosis, Thrombosis, and Vascular Biology 35, no. 9 (2015): 1963–1974, 10.1161/ATVBAHA.115.305896. PMC4548545 26139463

[36] M. Ma , W. Tang , A. Zhang , et al., “β‐Mangostin Triggers ER Stress‐Mediated Mitochondrial Dysfunction to Induce Apoptosis in Colorectal Cancer,” Phytomedicine 158 (2026): 158342, 10.1016/j.phymed.2026.158342. 42275878

[37] L. Li , N.‐N. Chen , Q.‐D. You , and X.‐L. Xu , “An Updated Patent Review of Anticancer Hsp90 Inhibitors (2013‐Present),” Expert Opinion on Therapeutic Patents 31, no. 1 (2021): 67–80, 10.1080/13543776.2021.1829595. 32990109

[38] J. Madrigal‐Matute , O. López‐Franco , L. M. Blanco‐Colio , et al., “Heat Shock Protein 90 Inhibitors Attenuate Inflammatory Responses in Atherosclerosis,” Cardiovascular Research 86, no. 2 (2010): 330–337, 10.1093/cvr/cvq046. 20154064

[39] H. Mu , L. Wang , and L. Zhao , “HSP90 Inhibition Suppresses Inflammatory Response and Reduces Carotid Atherosclerotic Plaque Formation in ApoE Mice,” Cardiovascular Therapeutics 35, no. 2 (2017): 891–921, 10.1111/1755-5922.12243. 28009484

[40] I. Lazaro , A. Oguiza , C. Recio , et al., “Targeting HSP90 Ameliorates Nephropathy and Atherosclerosis through Suppression of NF‐κB and STAT Signaling Pathways in Diabetic Mice,” Diabetes 64, no. 10 (2015): 3600–3613, 10.2337/db14-1926. 26116697

[41] X. Ding , C. Meng , H. Dong , et al., “Extracellular Hsp90α, Which Participates in Vascular Inflammation, Is a Novel Serum Predictor of Atherosclerosis in Type 2 Diabetes,” BMJ Open Diabetes Research & Care 10, no. 1 (2022): 002579, 10.1136/bmjdrc-2021-002579. PMC8804642 35091448

[42] S. Maiti and D. Picard , “Cytosolic Hsp90 Isoform‐Specific Functions and Clinical Significance,” Biomolecules 12, no. 9 (2022): 1166, 10.3390/biom12091166. PMC9496497 36139005

[43] F. H. Schopf , M. M. Biebl , and J. Buchner , “The HSP90 Chaperone Machinery,” Nature Reviews Molecular Cell Biology 18, no. 6 (2017): 345–360, 10.1038/nrm.2017.20. 28429788

[44] A. Rodina , C. Xu , C. S. Digwal , et al., “Systems‐Level Analyses of Protein‐Protein Interaction Network Dysfunctions via Epichaperomics Identify Cancer‐Specific Mechanisms of Stress Adaptation,” Nature Communications 14, no. 1 (2023): 3742, 10.1038/s41467-023-39241-7. PMC10290137 37353488

[45] P. Yan , H. J. Patel , S. Sharma , et al., “Molecular Stressors Engender Protein Connectivity Dysfunction Through Aberrant N‐Glycosylation of a Chaperone,” Cell Reports 31, no. 13 (2020): 107840, 10.1016/j.celrep.2020.107840. PMC7372946 32610141

[46] M. C. Inda , S. Joshi , T. Wang , et al., “The Epichaperome Is a Mediator of Toxic Hippocampal Stress and Leads to Protein Connectivity‐Based Dysfunction,” Nature Communications 11, no. 1 (2020): 319, 10.1038/s41467-019-14082-5. PMC6965647 31949159

[47] J.‐H. Lee and J. Lee , “Endoplasmic Reticulum (ER) Stress and Its Role in Pancreatic β‐Cell Dysfunction and Senescence in Type 2 Diabetes,” International Journal of Molecular Sciences 23, no. 9 (2022): 4843, 10.3390/ijms23094843. PMC9104816 35563231

[48] A. Salminen , K. Kaarniranta , and A. Kauppinen , “ER Stress Activates Immunosuppressive Network: Implications for Aging and Alzheimer's Disease,” Journal of Molecular Medicine 98, no. 5 (2020): 633–650, 10.1007/s00109-020-01904-z. PMC7220864 32279085

[49] M. L. Koloko Ngassie , C. A. Brandsma , R. Gosens , Y. S. Prakash , and J. K. Burgess , “The Stress of Lung Aging: Endoplasmic Reticulum and Senescence Tête‐à‐Tête,” Physiology 36, no. 3 (2021): 150–159, 10.1152/physiol.00039.2020. 33904785

[50] R. Zhang , M. Jiang , J. Zhang , et al., “Regulation of the Cerebrovascular Smooth Muscle Cell Phenotype by Mitochondrial Oxidative Injury and Endoplasmic Reticulum Stress in Simulated Microgravity Rats via the PERK‐eIF2α‐ATF4‐CHOP Pathway,” Biochimica et Biophysica Acta (BBA)—Molecular Basis of Disease 1866, no. 8 (2020): 165799, 10.1016/j.bbadis.2020.165799. 32304741

[51] M. Ketkar , S. Desai , P. Rana , et al., “Inhibition of PERK‐Mediated Unfolded Protein Response Acts as a Switch for Reversal of Residual Senescence and as Senolytic Therapy in Glioblastoma,” Neuro‐Oncology 26, no. 11 (2024): 2027–2043, 10.1093/neuonc/noae134. PMC11534322 39021199

[52] C. Anerillas , K. Mazan‐Mamczarz , A. B. Herman , et al., “The YAP–TEAD Complex Promotes Senescent Cell Survival by Lowering Endoplasmic Reticulum Stress,” Nature Aging 3, no. 10 (2023): 1237–1250, 10.1038/s43587-023-00480-4. PMC11369890 37667102

[53] T. Mikawa , M. Kameda , S. Ikari , et al., “Abrogation of Aberrant Glycolytic Interactions Eliminates Senescent Cells and Alleviates Aging‐Related Dysfunctions,” Signal Transduction and Targeted Therapy 10, no. 1 (2025): 402, 10.1038/s41392-025-02502-6. PMC12702999 41392167

[54] N. Sabath , F. Levy‐Adam , A. Younis , et al., “Cellular Proteostasis Decline in Human Senescence,” Proceedings of the National Academy of Sciences 117, no. 50 (2020): 31902–31913, 10.1073/pnas.2018138117. PMC7749315 33257563

[55] Y. Zhou , X. Wan , K. Seidel , et al., “Aging and Hypercholesterolemia Differentially Affect the Unfolded Protein Response in the Vasculature of ApoE−/− Mice,” Journal of the American Heart Association 10, no. 18 (2021): 020441, 10.1161/JAHA.120.020441. PMC8649520 34533042

[56] W. Ladiges , J. Morton , C. Blakely , and M. Gale , “Tissue Specific Expression of PKR Protein Kinase in Aging B6D2F1 Mice,” Mechanisms of Ageing and Development 114, no. 2 (2000): 123–132, 10.1016/s0047-6374(00)00097-x. 10799709

[57] X. Guan , Y. Hu , J. Hao , et al., “Stress, Vascular Smooth Muscle Cell Phenotype and Atherosclerosis: Novel Insight into Smooth Muscle Cell Phenotypic Transition in Atherosclerosis,” Current Atherosclerosis Reports 26, no. 8 (2024): 411–425, 10.1007/s11883-024-01220-8. 38814419

[58] P. Zhang , X. Yan , X. Zhang , et al., “TMEM215 Prevents Endothelial Cell Apoptosis in Vessel Regression by Blunting BIK‐Regulated ER‐to‐Mitochondrial Ca Influx,” Circulation Research 133, no. 9 (2023): 739–757, 10.1161/CIRCRESAHA.123.322686. 37750320

[59] G. Csordás , P. Várnai , T. Golenár , et al., “Imaging Interorganelle Contacts and Local Calcium Dynamics at the ER‐Mitochondrial Interface,” Molecular Cell 39, no. 1 (2010): 121–132, 10.1016/j.molcel.2010.06.029. PMC3178184 20603080

[60] S. Li , J. Chen , M. Liu , et al., “Protective Effect of HINT2 on Mitochondrial Function via Repressing MCU Complex Activation Attenuates Cardiac Microvascular Ischemia‐Reperfusion Injury,” Basic Research in Cardiology 116, no. 1 (2021): 65, 10.1007/s00395-021-00905-4. PMC8677646 34914018

[61] P. Pinton , A. Rimessi , S. Marchi , et al., “Protein Kinase C ß and Prolyl Isomerase 1 Regulate Mitochondrial Effects of the Life‐Span Determinant p66 Shc,” Science 315, no. 5812 (2007): 659–663, 10.1126/science.1135380. 17272725

[62] C. Hetz and F. R. Papa , “The Unfolded Protein Response and Cell Fate Control,” Molecular Cell 69, no. 2 (2018): 169–181, 10.1016/j.molcel.2017.06.017. 29107536

[63] Y. Gao , F. Ye , Y. Dong , et al., “Salvianic Acid A Ameliorates Atherosclerosis Through Metabolic‐Dependent Anti‐EndMT Pathway and Repression of TGF‐β/ALK5 Signaling,” Phytomedicine 136 (2025): 156307, 10.1016/j.phymed.2024.156307. 39740380

[64] L. Guarente , D. A. Sinclair , and G. Kroemer , “Human Trials Exploring Anti‐Aging Medicines,” Cell Metabolism 36, no. 2 (2024): 354–376, 10.1016/j.cmet.2023.12.007. 38181790

[65] S. Atlante , L. Cis , D. Pirolli , et al., “A Xanthine Derivative With Novel Heat Shock Protein 90‐Alpha Inhibitory and Senolytic Properties,” Aging Cell 24, no. 7 (2025): 70047, 10.1111/acel.70047. PMC12266748 40098059

[66] S. Zumerle , M. Sarill , M. Saponaro , et al., “Targeting Senescence Induced by Age or Chemotherapy With a Polyphenol‐Rich Natural Extract Improves Longevity and Healthspan in Mice,” Nature Aging 4, no. 9 (2024): 1231–1248, 10.1038/s43587-024-00663-7. PMC11408255 38951692

[67] S. O. Kocakaya , A. Ertas , I. Yener , et al., “Selective in‐Vitro Enzymes′ Inhibitory Activities of Fingerprints Compounds of Salvia Species and Molecular Docking Simulations,” Iranian Journal of Pharmaceutical Research: IJPR 19, no. 2 (2020): 187–198, 10.22037/ijpr.2020.112498.13801. PMC7667546 33224224

