# Supplementary material for: Salvianolic Acid a Disrupts the HSP90α‐AKT‐PERK Ternary Complex to Alleviate Atherosclerosis by Activating Endoplasmic Reticulum Stress of Senescent Vascular Smooth Muscle Cells
Source: Adv Sci (Weinh). 2026 Sep 27:e77891. Online ahead of print. doi: 10.1002/advs.77891 (PMC13616349; doi:10.1002/advs.77891)
Supplement: Supplementary file 1 [file ADVS-9999-e77891-s001.docx]

**Supplemental Table Ⅰ Search Strategy for Anti-tumor Natural Products.**

| #1 | ("Medicine, Chinese Traditional" OR "Zhong Yi Xue" OR "Chung I Hsueh" OR "Hsueh, Chung I" OR "Traditional Medicine, Chinese" OR "Chinese Traditional Medicine" OR "Traditional Chinese Medicine" OR "Chinese Medicine, Traditional") |
| --- | --- |
| #2 | ("Plants, Medicinal" OR "Medicinal Plant" OR "Plant, Medicinal" OR "Medicinal Plants" OR "Medicinal Herbs" OR "Herb, Medicinal" OR "Medicinal Herb" OR "Herbs, Medicinal" OR "Pharmaceutical Plants" OR "Pharmaceutical Plant" OR "Plant, Pharmaceutical" OR "Plants, Pharmaceutical" OR "Healing Plants" OR "Healing Plant" OR "Plant, Healing" OR "Plants, Healing" OR "Herb") |
| #3 | (“Tumors” OR “Neoplasia” OR “Neoplasias” OR “Neoplasm” OR “Tumor” OR “Cancer” OR “Cancers” OR “Malignant Neoplasm” OR “Malignancy” OR “Malignancies” OR “Malignant Neoplasms” OR “Neoplasm, Malignant” OR “Neoplasms, Malignant” OR “Benign Neoplasms” OR “Neoplasms, Benign” OR “Neoplasm, Benign” OR “Benign Neoplasm”) |
| #1 OR #2 | ("Medicine, Chinese Traditional" OR "Zhong Yi Xue" OR "Chung I Hsueh" OR "Hsueh, Chung I" OR "Traditional Medicine, Chinese" OR "Chinese Traditional Medicine" OR "Traditional Chinese Medicine" OR "Chinese Medicine, Traditional") OR ("Plants, Medicinal" OR "Medicinal Plant" OR "Plant, Medicinal" OR "Medicinal Plants" OR "Medicinal Herbs" OR "Herb, Medicinal" OR "Medicinal Herb" OR "Herbs, Medicinal" OR "Pharmaceutical Plants" OR "Pharmaceutical Plant" OR "Plant, Pharmaceutical" OR "Plants, Pharmaceutical" OR "Healing Plants" OR "Healing Plant" OR "Plant, Healing" OR "Plants, Healing" OR "Herb") |
| (#1 OR #2) AND #3 | (("Medicine, Chinese Traditional" OR "Zhong Yi Xue" OR "Chung I Hsueh" OR "Hsueh, Chung I" OR "Traditional Medicine, Chinese" OR "Chinese Traditional Medicine" OR "Traditional Chinese Medicine" OR "Chinese Medicine, Traditional") OR ("Plants, Medicinal" OR "Medicinal Plant" OR "Plant, Medicinal" OR "Medicinal Plants" OR "Medicinal Herbs" OR "Herb, Medicinal" OR "Medicinal Herb" OR "Herbs, Medicinal" OR "Pharmaceutical Plants" OR "Pharmaceutical Plant" OR "Plant, Pharmaceutical" OR "Plants, Pharmaceutical" OR "Healing Plants" OR "Healing Plant" OR "Plant, Healing" OR "Plants, Healing" OR "Herb")) AND (“Tumors” OR “Neoplasia” OR “Neoplasias” OR “Neoplasm” OR “Tumor” OR “Cancer” OR “Cancers” OR “Malignant Neoplasm” OR “Malignancy” OR “Malignancies” OR “Malignant Neoplasms” OR “Neoplasm, Malignant” OR “Neoplasms, Malignant” OR “Benign Neoplasms” OR “Neoplasms, Benign” OR “Neoplasm, Benign” OR “Benign Neoplasm”) |

**Supplemental Table Ⅱ Search Strategy for Anti-atherosclerotic Natural Products.**

| #1 | ("Medicine, Chinese Traditional" OR "Zhong Yi Xue" OR "Chung I Hsueh" OR "Hsueh, Chung I" OR "Traditional Medicine, Chinese" OR "Chinese Traditional Medicine" OR "Traditional Chinese Medicine" OR "Chinese Medicine, Traditional") |
| --- | --- |
| #2 | ("Plants, Medicinal" OR "Medicinal Plant" OR "Plant, Medicinal" OR "Medicinal Plants" OR "Medicinal Herbs" OR "Herb, Medicinal" OR "Medicinal Herb" OR "Herbs, Medicinal" OR "Pharmaceutical Plants" OR "Pharmaceutical Plant" OR "Plant, Pharmaceutical" OR "Plants, Pharmaceutical" OR "Healing Plants" OR "Healing Plant" OR "Plant, Healing" OR "Plants, Healing" OR "Herb") |
| #3 | (“Atherosclerosis” OR “Atheroscleroses” OR “Atherogenesis” OR “Atherogeneses”) |
| #1 OR #2 | (“Coronary Artery Disease” OR “Artery Disease, Coronary” OR “Artery Diseases, Coronary” OR “Coronary Artery Diseases” OR “Coronary Arteriosclerosis” OR “Arterioscleroses, Coronary” OR “Coronary Arterioscleroses” OR “Arteriosclerosis, Coronary” OR “Atherosclerosis, Coronary” OR “Atheroscleroses, Coronary” OR “Coronary Atheroscleroses” OR “Coronary Atherosclerosis” OR “Left Main Coronary Artery Disease” OR “Left Main Coronary Disease” OR “Left Main Disease” OR “Left Main Diseases”) |
| (#1 OR #2) AND #3 | (“Coronary Disease” OR “Coronary Diseases” OR “Disease, Coronary” OR “Diseases, Coronary” OR “Coronary Heart Disease” OR “Coronary Heart Diseases” OR “Disease, Coronary Heart” OR “Diseases, Coronary Heart” OR “Heart Disease, Coronary” OR “Heart Diseases, Coronary”) |

**Supplemental Table Ⅲ Single-Cell Transcriptome Samples.**

| **Datasets** | **GEO Accession** | **Sample size** | **Tissue** | **Ref.** |
| --- | --- | --- | --- | --- |
| Pan | GSE155514 | 3 | carotid artery | ^1^ |
| Alsaigh | GSE159677 | 6 | carotid artery | ^2^ |
| Bashore | GSE253903 | 12 | carotid artery | ^3^ |
| Fidler | GSE247238 | 19 | carotid artery | ^4^ |

**Supplemental Fig. Ⅰ Western Blot Analysis.**

**Supplemental Fig. Ⅰ** (A) Western blot analysis of HSP90α knockdown. (B) Western blot analysis of PERK knockdown. (C) Western blot analysis of HSP90α overexpression. (D) Western blot analysis of MCU knockdown. (E) Quantification of the Western blot data from (A–D). n = 3. ^**^*P* < 0.01; ^***^*P* < 0.001.

**Supplemental Fig. Ⅱ Western Blot Analysis of ATF6 and IR1E signaling.**

**Supplemental Fig.** **Ⅲ Dose-response curves of thapsigargin-treated normal and senescent VSMCs.**

**Supplemental Fig. Ⅳ** **Western blot analysis following PERK knockdown**

**Supplemental Fig. Ⅳ** (A) Western blot analysis of p16 and p21 protein levels following PERK knockdown; (B) Western blot detection of SM-MHC, α-SMA and CD68 protein expression upon PERK knockdown; (C) Western blot examination of collagen I protein expression after PERK knockdown.

**Supplemental Fig. Ⅴ The domain mapping results**

**Supplemental Fig. Ⅵ ER‑mitochondria contacts labeled by fluorescent probes**

**Supplemental Fig. Ⅶ The levels of SASP in senescent VSMCs cultures**

**Supplemental Fig. Ⅷ Western blot analysis following SAA treatment**

**Supplemental Fig. Ⅸ SAA have no pro-apoptotic effect to normal VSMCs**

**Supplemental Fig. Ⅸ** (A) The CCK8 results; (B) Mito-Tracker Red CMXRos and Annexin V-FITC staining of normal VSMCs pretreated with SAA.

**Supplemental Fig.** **Ⅹ** **Pull-down results**

**Supplemental Fig. Ⅺ Western blot analysis of PERK signaling pathway in normal VSMCs**

**Supplemental Fig. Ⅺ** (A) Western blot analysis of PERK activation in normal VSMCs; (B) Western blot analysis of eIF2α activation in normal VSMCs; (C) Western blot analysis of ATF4-CHOP activation in normal VSMCs.

**Supplemental Fig. Ⅻ** **The Cross-linking results**

**Supplemental Fig. ⅩⅢ ER‑mitochondria contacts labeled by fluorescent probes**

**Supplemental Fig. ⅩⅣ ER‑mitochondria contacts labeled by fluorescent probes**

**Supplemental Fig. ⅩⅤ Mito-Tracker Red CMXRos and Annexin V-FITC staining**

**Supplemental Fig. ⅩⅥ The IL-6 level**

**Supplemental Fig. ⅩⅦ Quantification of immunofluorescence intensity of p21**
